# Automated Machine Learning Analysis of Patients With Chronic Skin Disease Using a Medical Smartphone App: Retrospective Study

**DOI:** 10.2196/50886

**Published:** 2023-11-28

**Authors:** Igor Bibi, Daniel Schaffert, Mara Blauth, Christian Lull, Jan Alwin von Ahnen, Georg Gross, Wanja Alexander Weigandt, Johannes Knitza, Sebastian Kuhn, Johannes Benecke, Jan Leipe, Astrid Schmieder, Victor Olsavszky

**Affiliations:** 1 Department of Dermatology, Venereology and Allergology University Medical Center and Medical Faculty Mannheim Center of Excellence in Dermatology, Heidelberg University Mannheim Germany; 2 Department of Medicine V, Division of Rheumatology University Medical Centre and Medical Faculty Mannheim Heidelberg University Mannheim Germany; 3 Institute of Digital Medicine Philipps-University Marburg and University Hospital of Giessen and Marburg Marburg Germany; 4 Department of Dermatology, Venereology, and Allergology University Hospital Würzburg Würzburg Germany

**Keywords:** automated machine learning, psoriasis, hand and foot eczema, medical smartphone app, application, smartphone, machine learning, digitalization, skin, skin disease, use, hand, foot, mobile phone

## Abstract

**Background:**

Rapid digitalization in health care has led to the adoption of digital technologies; however, limited trust in internet-based health decisions and the need for technical personnel hinder the use of smartphones and machine learning applications. To address this, automated machine learning (AutoML) is a promising tool that can empower health care professionals to enhance the effectiveness of mobile health apps.

**Objective:**

We used AutoML to analyze data from clinical studies involving patients with chronic hand and/or foot eczema or psoriasis vulgaris who used a smartphone monitoring app. The analysis focused on itching, pain, Dermatology Life Quality Index (DLQI) development, and app use.

**Methods:**

After extensive data set preparation, which consisted of combining 3 primary data sets by extracting common features and by computing new features, a new pseudonymized secondary data set with a total of 368 patients was created. Next, multiple machine learning classification models were built during AutoML processing, with the most accurate models ultimately selected for further data set analysis.

**Results:**

Itching development for 6 months was accurately modeled using the light gradient boosted trees classifier model (log loss: 0.9302 for validation, 1.0193 for cross-validation, and 0.9167 for holdout). Pain development for 6 months was assessed using the random forest classifier model (log loss: 1.1799 for validation, 1.1561 for cross-validation, and 1.0976 for holdout). Then, the random forest classifier model (log loss: 1.3670 for validation, 1.4354 for cross-validation, and 1.3974 for holdout) was used again to estimate the DLQI development for 6 months. Finally, app use was analyzed using an elastic net blender model (area under the curve: 0.6567 for validation, 0.6207 for cross-validation, and 0.7232 for holdout). Influential feature correlations were identified, including BMI, age, disease activity, DLQI, and Hospital Anxiety and Depression Scale-Anxiety scores at follow-up. App use increased with BMI >35, was less common in patients aged >47 years and those aged 23 to 31 years, and was more common in those with higher disease activity. A Hospital Anxiety and Depression Scale-Anxiety score >8 had a slightly positive effect on app use.

**Conclusions:**

This study provides valuable insights into the relationship between data characteristics and targeted outcomes in patients with chronic eczema or psoriasis, highlighting the potential of smartphone and AutoML techniques in improving chronic disease management and patient care.

## Introduction

### Background

The process of digitalization of the world’s economic and social systems has been advancing at an increasing rate in recent years. In addition, the COVID-19 pandemic acted as a catalyst for the digital transformation of industries and businesses [1]. New digital technologies have inevitably affected the health care sector as well. Telemedicine is being implemented to exchange medical data remotely [2], telemonitoring facilitates real-time observations of patients who are bedridden or chronically ill [3], and video consultation quickly became a necessity during the COVID-19 lockdown [4]. Moreover, smartphones have emerged as the main communication device worldwide [5], with computing capabilities that go beyond the scope of a simple phone call or SMS text message. Their implementation in health care systems has been termed mobile health (mHealth) by the World Health Organization [6] and has already provided growing evidence of improvements in health outcomes and health services [7-9].

For the past decade, Germany has made considerable efforts to digitalize its health care system [10]. For this purpose, the German parliament passed the Digital Healthcare Act (ie, *Digitale-Versorgung-Gesetz*) on November 7, 2019, allowing physicians to prescribe digital health applications, which are reimbursed by statutory health insurers [11]. However, a recent nationwide cross-sectional survey showed that the actual use of digital health applications in Germany is not widespread and that users’ trust in internet-based health decisions is low [12]. Furthermore, there is a scarcity of health care app use in certain German health care sectors such as psychiatry [13]. A possible explanation for the lower use of mHealth apps among patients could be their unknown effectiveness and the lack of high-quality studies [14]. We have previously shown that patients with psoriasis undergoing an educational program combined with a monitoring smartphone app had a significantly greater reduction in depression and anxiety symptoms when using the app less than once a month [15]. Notably, the long-term effects observed for >60 weeks in the same clinical trial showed similar significant reductions in depression and anxiety [16]. As the educational program alone did not show any influence on these psychological symptoms [17], the use of the monitoring app provided an additional benefit to the mental health of patients with psoriasis. This is particularly relevant given that psychiatric comorbidities are highly prevalent in chronic diseases such as psoriasis [18,19].

Psoriasis is a chronic inflammatory skin disease that has systemic pathological effects and is associated with psoriatic arthritis in almost 30% of the cases [20]. As there is no curative therapy for psoriasis, various treatments are used to control its symptoms, which can be disabling in some cases [21]. Moreover, psoriasis is known to heavily affect patients’ health-related quality of life (QoL) [22], which may be exacerbated by comorbidities [23]. Another distinct yet similarly burdensome chronic inflammatory skin disease is atopic dermatitis, which is characterized by pruritus and skin barrier dysfunction. As psoriasis and atopic dermatitis share common features such as immune cell infiltration of the skin with overexpression of proinflammatory cytokines, genetic predisposition triggered by environmental factors, and having a negative impact on individuals and society, they are often discussed together by clinicians and epidemiologists [24,25]. Despite recent advances in the development of both topical and systemic therapies for psoriasis and eczema [26,27], these 2 conditions remain the most common chronic skin diseases worldwide, with a combined prevalence of 3% to 10% and rising [25]. Knowing that smartphone apps have emerged as effective tools in the management of chronic diseases [28], the implementation of further digital technologies could help to treat chronic psoriasis and eczema.

Another area of technology that is increasingly being used in health care is machine learning (ML), a subdiscipline of artificial intelligence [29]. ML trains a predictive computational model by recognizing patterns in data and then using that model to make predictions. ML applications in health care include forecasting disease progression and mortality, classifying diseases from clinical images, or interpreting genomic data [30]. Despite its diagnostic and predictive capabilities, ML still relies on data scientists to perform complex tasks that facilitate ML analysis. To name a few, these tasks include data set preparation, selection of a single appropriate computational model, optimization of hyperparameters, or postprocessing of the selected model. As a result, the growing demand for ML applications cannot be met by non–data scientists [31]. This problem has been addressed using a novel, user-friendly ML technology called automated ML (AutoML) [32]. AutoML enables researchers without extensive coding or data science expertise to rapidly build superior predictive models by performing massive parallel processing. Given that health care systems generate extensive amounts of so-called big data sets [33,34], AutoML serves as an ideal tool to quickly analyze big data and build predictive models that clinics can use to reduce costs and improve patient care. We have recently performed forecasting of several diseases using AutoML on an *International Classification of Diseases, Tenth Revision* (ICD-10) database of an entire European country [35,36].

ML approaches have been applied extensively in dermatology. The main area of ML application in dermatology is disease classification using clinical or histopathological images [37]. Skin cancer data, especially melanoma data, are mainly used to train ML algorithms to detect these malignant lesions at an early stage. Notably, patients seem to accept ML-based applications for melanoma diagnosis, whereas there are no data on the acceptance of ML for melanoma diagnosis among clinicians [38]. Conversely, artificial intelligence–based classification of histopathological melanoma images has been met with criticism by practicing clinicians because of cropped, unsuitable images or omission of borderline lesions [39]. Regarding chronic skin diseases, systematic reviews of the current literature have identified 2 main ML use cases in psoriasis, namely, skin image evaluation and complication or treatment prediction [40], whereas in atopic dermatitis, ML has been used primarily for genomic data set analysis [41].

### Objectives

In this study, we performed an AutoML analysis of a clinical data set of patients with psoriasis and hand and foot dermatitis who used a medical smartphone app during interventional studies conducted at our university hospital from 2018 to 2021. The aim of this study was to gain new insights into the activity and treatment response of these chronic diseases by considering different variables such as comorbidities, medical scores, and the use of a monitoring smartphone app. To our knowledge, this is the first study to apply the novel ML tool, AutoML, to a dermatological mHealth data set.

## Methods

### Data Selection

Three separate primary data sets were generated from clinical trials of patients with chronic inflammatory skin conditions undergoing an educational program combined with a smartphone monitoring app [15,16]. The use of the smartphone app was expected weekly with the intention of documenting disease activity via patient-generated images of the skin and completion of a patient questionnaire including the numeric rating scale (NRS) for pain and itching and the Dermatology Life Quality Index (DLQI). It should be noted that pain, itching, and DLQI scores were also additionally documented by the investigators at baseline and at follow-up visits.

**Figure 1:**
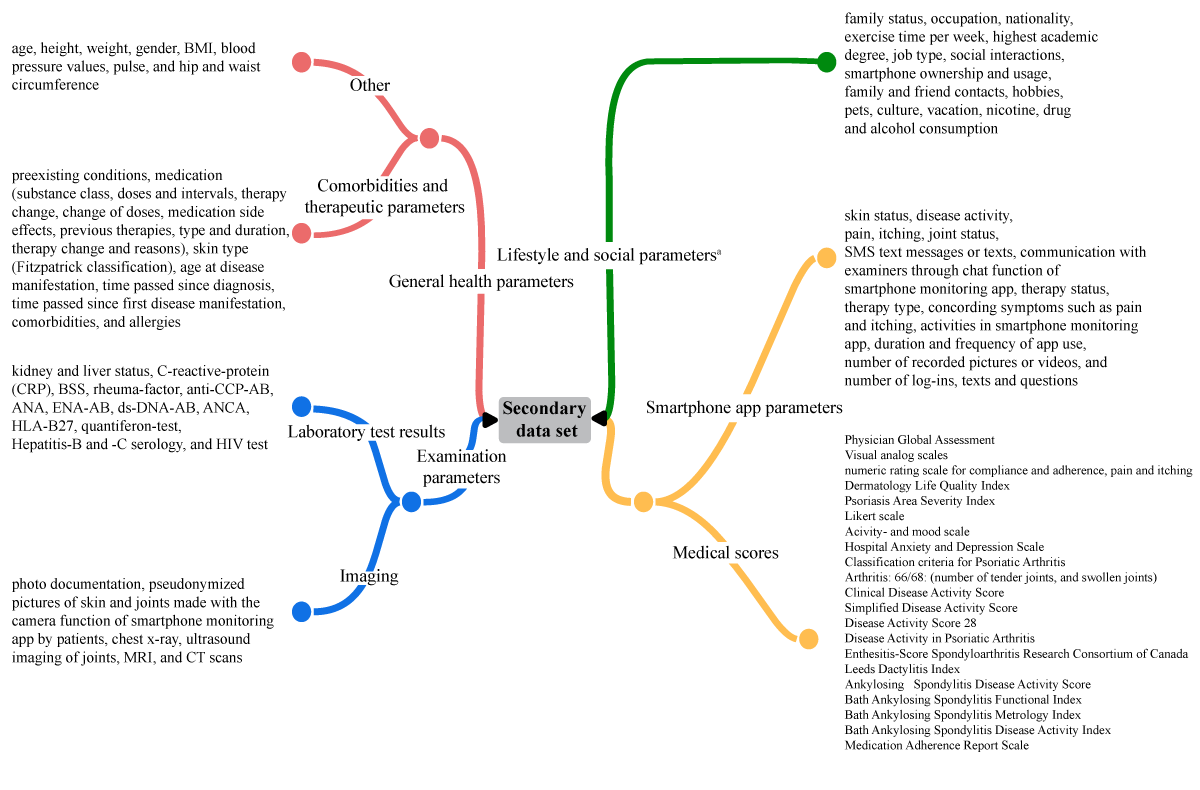
General overview of patient characteristics (features) of the 3 primary data sets categorized into different parameter groups. Patient features were grouped into general health parameters, examination parameters, lifestyle and social parameters, smartphone app parameters, and medical scores. Only common features in all 3 primary data sets were extracted to create a secondary data set of patients with psoriasis vulgaris and chronic hand and/or foot eczema.

The first 2 studies were conducted between 2018 and 2020 and included 107 patients with psoriasis vulgaris and 99 patients with atopic and chronic hand and/or foot eczema. Their primary data sets consisted of 135 and 88 different patient characteristics, respectively, that is, features, including general health parameters (eg, age, height, and weight); comorbidities and therapeutic parameters (eg, preexisting conditions and medications); laboratory test results (eg, renal and liver status and C-reactive protein level); imaging (eg, photo documentation); lifestyle and social parameters (eg, marital status, occupation, nationality, and sports); smartphone app parameters (eg, skin status, disease activity, pain, and itching); and medical scores (eg, Physician Global Assessment, Visual Analog Scale, NRS, and DLQI; Figure 1). Follow-up visits were conducted at 3, 6, 9, and 15 months after the start of the study. A third clinical trial using the same smartphone monitoring app was conducted from 2020 to 2021 with 202 participants, predominantly those with both psoriasis vulgaris and psoriatic arthritis. These patients were evaluated at 3 different visits: at study onset, at 3 months, and at 6 months. Similar patient data were also collected, including psoriatic arthritis parameters, resulting in a total of 552 features. All 3 studies were conducted in our Department of Dermatology by the same team of clinical investigators.

### Data Preparation and Extraction

Given the identical conditions and similar design of the abovementioned clinical trials, we extracted and merged all common features from their primary data sets to create a new secondary data set with patients with both psoriasis vulgaris and hand and/or foot eczema (Figure 1). The newly combined secondary data set consisted of 368 patients with 67 different features. Patients lost to follow-up or those with incomplete data set features were excluded. Furthermore, new features were created to enrich the data set and gain further insight into the course of the clinical studies, therapy efficacies, and health effects of the smartphone monitoring app used. Specifically, new general health parameters were added by calculating BMI, physical activity level according to patients’ job type, and exercise time per week (Multimedia Appendices 1 and 2). With regard to medical scores, the development of pain and itching, DLQI, Hospital Anxiety and Depression (HADS)–Anxiety (HADS-A) score, and HADS-Depression (HADS-D) score between study inclusion and the 6-month follow-up visit were also calculated. Pain and itching status development was defined as “reduction of itching or pain,” “consistently itch free or pain free,” “became free of itching,” “constant low level of itching or pain,” “increase in itching or pain,” and “constant severe itching or pain” (Multimedia Appendix 2). DLQI development after 6 months was categorized as “improved QoL,” “consistently best QoL,” “consistently good QoL,” “consistently mediocre QoL,” “reduction in QoL,” and “consistently poor QoL.” In addition, the DLQI at onset and at 3-month and 6-month follow-up, together with HADS-A and HADS-D, were further reclassified as categorical features with predefined cutoff values (Multimedia Appendix 2). Images and blood test results were excluded owing to high heterogeneity. Other intentionally excluded features are the Psoriasis Area and Severity Index and Hand Eczema Severity Index scores, which are highly specific for one or the other disease. Most rheumatic scores, which only represent joint involvement in psoriatic arthritis, were also excluded. Such scores include the Bath Ankylosing Spondylitis Disease Activity Index, Classification for Psoriatic Arthritis, Ankylosing Spondylitis Disease Activity Score, and Clinical Disease Activity Index. Finally, new smartphone app parameters were also extracted and calculated from the app logs, including average pain, itching, DLQI, and HADS ratings, hereafter referred to as “app average pain,” “app average itching,” “app average DLQI,” and “app average mood” (Multimedia Appendices 1 and 2). The data were not normalized during data preparation. This pseudonymized secondary data set, containing only common and newly computed features, was used for further AutoML analysis.

### Experimental Setup and Exploratory Data Analyses

After extensive data preparation, the secondary clinical data set was imported into the DataRobot AutoML platform [36,42]. The AutoML platform performs an initial exploratory data analysis to summarize the data set’s main characteristics and to automatically create feature transformations. The automatic feature transformations do not replace the raw features but categorize them into different types (eg, numeric, categorical, Boolean, date, currency, and percentage). Moreover, numerical statistics such as mean, SD, median, minimum, and maximum are provided for the numerical features, and the frequency distribution is shown for the top 50 items of each feature (Multimedia Appendix 3). Then, a target is selected, and a second exploratory data analysis is performed for the recalculation of the numerical statistics, feature correlation with the target, and model building. Additional data quality issues are identified, including outliers, multicategorical format errors, inliers, excess zeros, hidden missing values, and target leakage. A report on the handling of data quality, including the processing of missing values, is provided in Multimedia Appendix 3. Outliers are identified using the algorithm by Ueda [43], whereas target leakages are either calculated and flagged for the user to check or automatically removed if they exceed a certain threshold (Multimedia Appendix 3).

For this study, 4 different targets were selected from the clinical data set’s features list. The selected targets were “itching development for 6 months,” “pain development for 6 months,” “DLQI development for 6 months,” and “app use.” Then, data quality was manually improved according to the exploratory data analyses results. Redundant features and data leakage were removed, resulting in the final list of features used for modeling (Multimedia Appendix 4). As 147 (39.9%) of the 368 total patients lacked 3-month follow-up data, we excluded this time point for the targets “itching development for 6 months,” “pain development for 6 months,” and “DLQI development for 6 months” to avoid overemphasis and model building based on missing values by the AutoML platform. This exclusion was because of the design of the trials, which meant that not all patients were seen at the 3-month follow-up visit, unless they specifically requested a follow-up appointment or experienced a worsening of their condition that required a further intervention in their therapy. Feature types were changed if they were incorrectly categorized by the AutoML platform. Moreover, once the target is selected, DataRobot automatically determines the type of analysis based on the target’s feature type. A regression analysis is performed if the target is a numerical value, or a classification analysis is performed in case of a categorical target. Finally, all the conditions are met to start a massive parallel modeling process that will ultimately allow the selection of the most accurate ML model.

### Model Selection and Documentation

During the modeling process, the AutoML platform computes the optimum model for a certain target through countless combinations of data transformations. It automatically ranks models based on procedures such as boosting, bagging, random forests, kernel-based methods, generalized linear models, and deep learning. Blenders can improve the model performance or produce even more accurate versions of the superior ranking models. To train, validate, and rank a model, the data set is partitioned into training, validation, and holdout sets. For multiclass targets 1 to 3, modeling partitions were created using random sampling. In contrast, the partitions for the binary classification in target 4 were selected using a stratified sampling to preserve the distribution of the target for each partition. The training data segment, approximately 65% of the data set, was used to build ML models that uncover the relationships between the target and all other features. The validation split, approximately 15% of the data set, was used to test the accuracy of the model. Once automated modeling was complete, all ML models were ranked according to their scores on the platform’s leaderboard. The scores listed were validation, cross-validation (CV), and holdout scores. CV represents the mean of 5 scores calculated on 5 different partitions of the training and validation splits. Specifically, the nonholdout data are divided into smaller partitions called “folds.” The AutoML platform first trains models on a smaller portion of the data and uses only 1 CV fold to evaluate model performance. It then trains only the best models on the full CV partitions. For these models, the AutoML platform performs *k*-fold (eg, 5-fold) CV training and evaluation. Specifically, in each iteration of the model building, 4 of the 5 partitions were used to train the model, and the last 1 was reserved for validation. This process was repeated 5 times, each time switching to the next partition to be used for validation and the remainder to be used for training (Multimedia Appendix 5). The final CV scores are the average scores of each CV fold performance. The holdout segment, by contrast, comprises approximately 20% of the data set, is completely separate from the validation and training partitions, and is not used during the training and validation processes. It can be used as a final estimate of the performance of an ML model. In this study, the logarithmic (log) loss metric, or cross-entropy loss, was primarily considered as an accuracy score when selecting the best-performing model for multiclass classification, as with targets 1 to 3, as its advantages lie in its suitability for evaluating imbalanced data sets [44,45]. This performance metric is considered an appropriate and widely used evaluation metric for ML applications [46]. For binary classification, as was the case for target 4, we considered the area under the receiver operating characteristic (ROC) curve as the main performance metric for a more accessible interpretation. ROC area under the curve (AUC) values are provided for multiclass models as macroaverages from each class, weighted by the number of true instances for each class. Other calculated estimators included accuracy, fraction of variance explained (FVE) multinomial or binomial, *F*_1_-score, recall, and precision.

**Figure 2:**
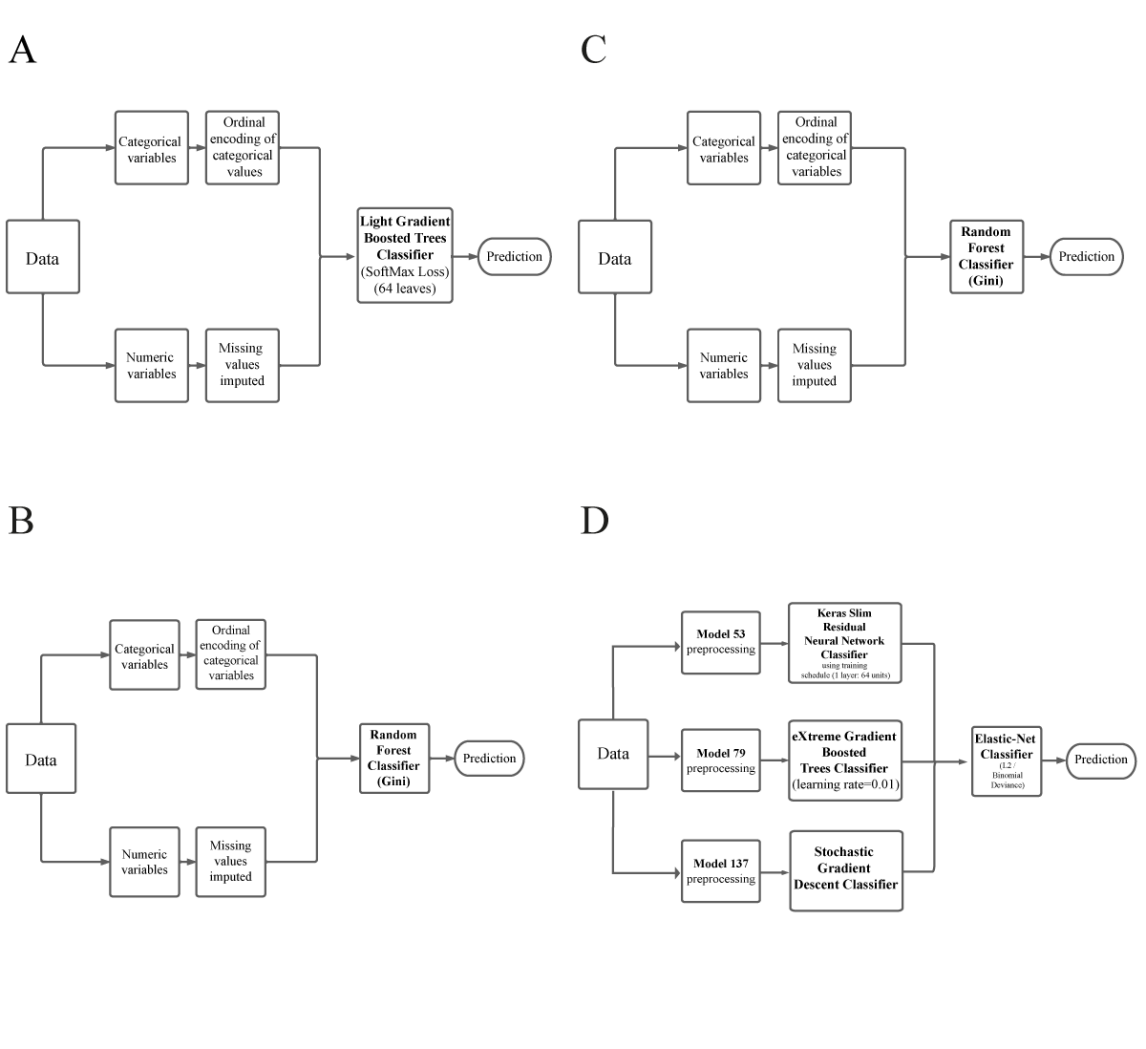
Model development workflow process (model blueprint) for selected targets: (A) "itching development for 6 months," (B) "pain development for 6 months," (C) "DLQI development for 6 months," and (D) "app use." During modeling process, the AutoML platform performs a variety of feature engineering combinations, preprocessing techniques and machine learning algorithms to uncover relationships and patterns between the selected target and the data set's features. The graphic depicts the numerous steps necessary to convert input predictors and targets into a model. Each blueprint node can represent multiple processing steps. AutoML: automated machine learning; DLQI: Dermatology Life Quality Index.

To provide insight into the modeling process, DataRobot produces a model-specific documentation including a blueprint. The blueprints contain all the preprocessing steps, modeling algorithms, and postprocessing steps that have been performed during model building. Figure 2 shows a graphical layout of the ML model selected for each target.

### Ethical Considerations

This study was reviewed and approved by the Medical Ethics Committee II of the Medical Faculty Mannheim, Heidelberg University, Germany (2021-895). The 3 clinical trials conducted between 2018 and 2021, whose primary data sets were used to generate the secondary data set of this study, were approved by the same medical ethics committee and conducted in accordance with the principles of the Declaration of Helsinki (2017-655N-MA and 2020-515N-MA).

## Results

### Overview

The management of chronic skin diseases mainly consists of alleviating symptoms and ensuring a better QoL because cures are still not available despite recent biomedical advances. Therefore, after preparing and revising the secondary data set, we set out to identify critical relationships among its different data features to better understand how symptoms and QoL are influenced by other data set parameters. Therefore, we selected the development of itching and pain and the DLQI score for 6 months after study entry as AutoML targets. Given that our disease monitoring app, used in clinical trials for patients with psoriasis and hand and/or foot eczema, showed significant benefits in reducing depression and anxiety in patients with psoriasis [15,16] and improving QoL and pain measures in patients with eczema [47], we also chose the patient use of the app as another outcome to better understand the patients’ willingness to use the app and their compliance with using such a digital medical tool, as influenced by the different data set features.

A multiclass classification analysis was performed for the target “itching development for 6 months” (Figure 2A), training a total of 78 models. The model selected was the light gradient boosted trees classifier (SoftMax loss; 64 leaves), with a log loss of 0.9302 for the validation split, 1.0193 for CV split, and 0.9167 for the holdout split (Multimedia Appendix 6; Table 1).

A further multiclass classification analysis, training a total of 54 models, was performed for the target “pain development for 6 months” (Figure 2B). In this case, the model chosen was a random forest classifier (Gini). Its metric scores were a log loss of 1.1799 for validation, 1.1561 for CV, and 1.0976 for holdout, with a sample size of 100% (293/293; Multimedia Appendix 6; Table 2).

A total of 27 models were trained for the multiclass target “DLQI development for 6 months” (Figure 2C) with the chosen model also being a random forest classifier (Gini) with a sample size of 64.21% (192/299). In this case, the log loss was 1.3670 for validation, 1.4354 for CV, and 1.3974 for holdout (Multimedia Appendix 6; Table 3).

Finally, a binary classification analysis was performed for the target “app use” (Figure 2D), training a total of 216 models. The model chosen was an elastic net (ENET) blender consisting of the 3 best-performing models for AUC in the holdout partition, namely, the Keras slim residual neural network classifier using training schedule (1-layer: 64 units), the extreme gradient boosted trees classifier (learning rate=0.01), and the stochastic gradient descent classifier. This final model achieved an AUC score of 0.6567 for the validation split, 0.6207 for the CV split, and 0.7232 for the holdout split (Multimedia Appendix 6; Table 4). Learning curves provide a more detailed view of the performance metrics that were achieved during training (Multimedia Appendix 7).

**Table 1:** Accuracy metrics for the target “itching development for 6 months.” Logarithmic (log) loss, area under the curve (AUC), accuracy, and fraction of variance explained (FVE) multinomial scores in validation, cross-validation (CV), and holdout partitions are listed for the selected model.

Class name	Log loss	AUC	Accuracy	FVE multinomial
Validation	0.9302	0.8096	0.7234	0.2594
CV	1.0193	0.7748	0.6511	0.1642
Holdout	0.9167	0.6734	0.6897	0.0521

**Table 2:** Accuracy metrics for the target “pain development for 6 months.” Logarithmic (log) loss, area under the curve (AUC), accuracy, and fraction of variance explained (FVE) multinomial scores in validation, cross-validation (CV), and holdout partitions are listed for the selected model.

Class name	Log loss	AUC	Accuracy	FVE multinomial
Validation	1.1799	0.7545	0.4468	0.2047
CV	1.1561	0.7684	0.4596	0.2138
Hold out	1.0976	0.7622	0.5	0.1780

**Table 3:** Accuracy metrics for the target “Dermatology Life Quality Index development for 6 months.” Logarithmic (log) loss, area under the curve (AUC), accuracy, and fraction of variance explained (FVE) multinomial scores in validation, cross-validation (CV), and holdout partitions are listed for the selected model.

Class name	Log loss	AUC	Accuracy	FVE multinomial
Validation	1.4065	0.7072	0.3542	0.0799
CV	1.3650	0.7678	0.4250	0.1557
Holdout	1.4548	0.7260	0.4237	0.1477

**Table 4:** Accuracy metrics for the target “app use.” F1-score, recall, precision, logarithmic (log) loss, area under the curve (AUC), accuracy, and fraction of variance explained (FVE) binomial scores in validation, cross-validation (CV), and holdout partitions are listed for the selected model.

Partition	*F*_1_-score	Recall	Precision	Log loss	AUC	Accuracy	FVE binomial
Validation	0.6667	0.8214	0.561	0.6509	0.6567	0.6102	0.0592
CV	0.6552	0.9683	0.4963	0.6698	0.6207	0.5254	0.0305
Holdout	0.7179	0.8235	0.6364	0.6389	0.7232	0.6986	0.0751

### Target 1: Itching Development for 6 Months

**Figure 3:**
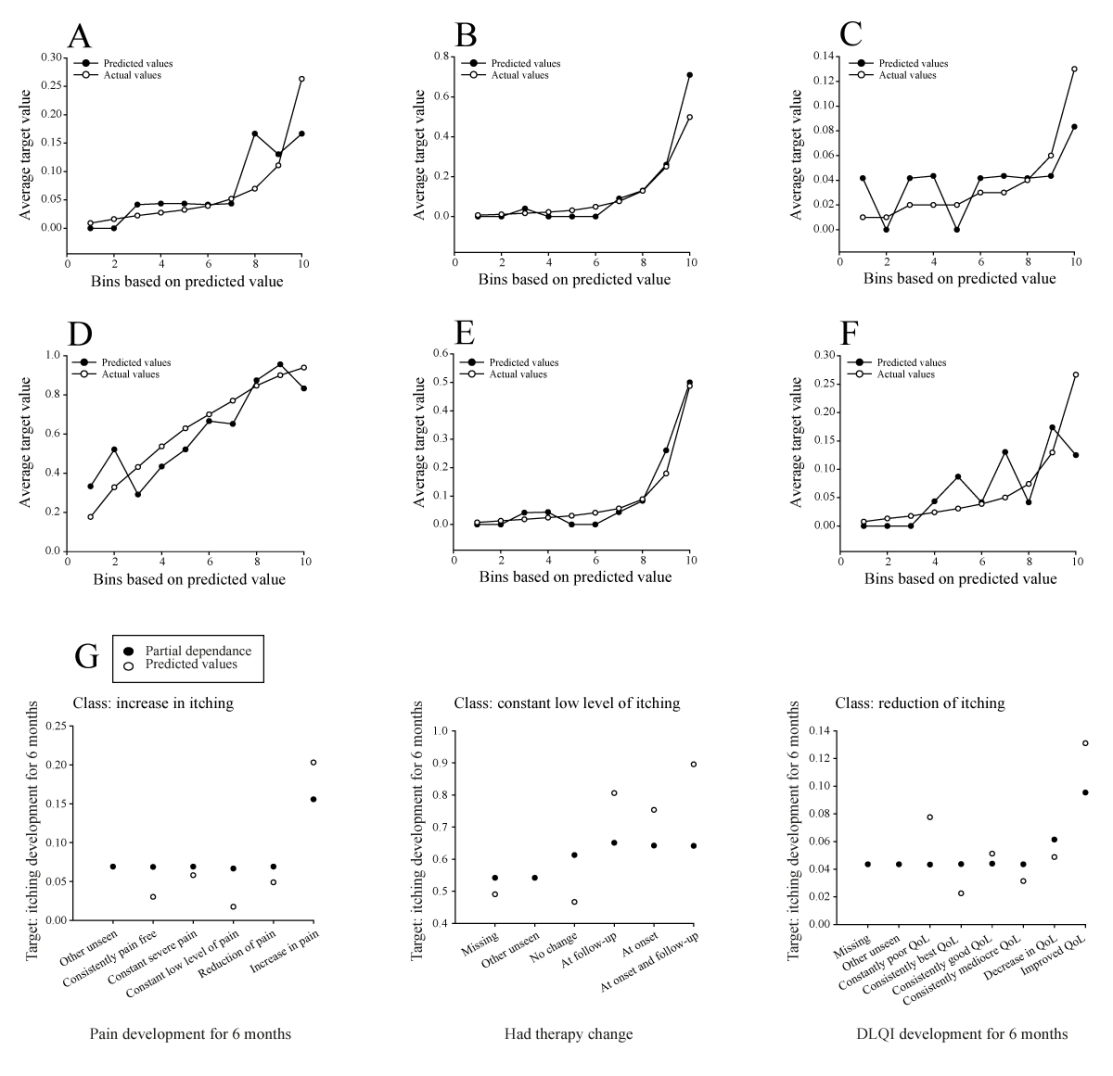
Evaluation of the selected model for the target "itching development for 6 months." Lift charts for all 6 target classes for CV partition: (A) reduction of itching, (B) consistently itch free, (C) became free of itching, (D) constant low level of itching, (E) increase in itching, and (F) constant severe itching. (G) The selection of feature effects of the features "pain development for 6 months" on the class "increase in itching," "had therapy change" on the class "constant low level of itching," and "DLQI development for 6 months" on the class "reduction of itching." All feature effects are shown for the validation partition. CV: cross-validation; DLQI: Dermatology Life Quality Index.

The performance of the selected light gradient boosted tree classifier (SoftMax loss) model with 64 leaves was demonstrated using lift charts (Figure 3A-F). The x-axis of the presented lift charts represents sorted and grouped numerical feature values in equal-sized bins, whereas the y-axis represents the lift, which is the ratio of the model’s performance. The points on the lift chart indicate the average percentage in each bin. The “predicted” values display the average prediction score for the rows in that bin, whereas the “actual” values show the average value of the data distribution within each bin. The higher the lift, the more effective the model is at identifying the target outcome. Another accuracy indicator was the closeness of the predicted line to the actual line.

The selected feature target “itching development for 6 months” represents a newly calculated feature from the secondary data set that was categorized into 6 different classes: reduction of itching, consistently itch free, became free of itching, constant low level of itching, increase in itching, and constant severe itching (Multimedia Appendix 2). Of the 6 subclasses, “constant low level of itching” had the highest proportion of distribution, with 60.85% (143/235) of the training partition attributed to this subclass. In total, 12.3% (29/235) were attributed to the “consistently itch free” class, 9.8% (23/235) to “increase in itching,” 6.8% (16/235) to “decrease in itching,” 6.4% (15/235) to “constant severe itching,” and finally 3.8% (9/235) to “became free of itching.” Considering the closeness of the predicted value lines to the corresponding actual value lines, our selected model shows the best accuracy for the classes “consistently itch free” and “increase in itching” (Figure 3B and E). This observation was further supported by the upward trajectories of the 2 curves. In addition, the model was relatively successful in identifying patients who were likely to experience a reduction in itching and a constant low level of itching after 6 months, as indicated by steadily increasing lifts (Figure 3A and D). Finally, the model showed a moderate ability to identify patients who will transition to an itch-free state after 6 months or patients who will continue to experience severe itching after 6 months (Figure 3C and F). Although the predicted values for these last 2 itching classes are somewhat scattered, there is still a positive trend in the curves, indicating that the model can predict the outcome to some extent but may not be as accurate as desired.

Feature Impact, a technique available for all model types, measures the effect of changes in the input training data on a model’s score. This approach, also known as permutation importance, assesses how much a model’s error score would worsen if predictions were made after randomly shuffling a particular column while leaving the other columns unchanged. The AutoML platform normalizes the scores. It assigns the highest value to the most influential feature column and normalizes the other features accordingly. Part A in Multimedia Appendix 8 shows the top 10 most influential aggregated features for all target classes, of which “had therapy change” had the strongest impact, normalized to 1.00. These features can be used to better understand the factors influencing the model’s predictions of itching development after 6 months in patients with psoriasis and atopic dermatitis. Another important feature impact in the aggregated chart was the DLQI score at follow-up with an impact of 0.61 and onset disease activity with an impact of 0.53.

The feature effects (Multimedia Appendix 9) demonstrate how changes in the value of each feature affect the model’s predictions, with partial dependence plots providing insights into the relationship between each feature and the target outcome. A partial dependency plot shows the change in the target predictions when a specific feature is altered while all other features remain constant. For example, a selection of 3 specific features and their effects on different classes of itching development after 6 months (Figure 3G) showed that if there is no change in therapy during the trial, the probability of maintaining a constant low level of itching is lower. This was further supported by the model’s predicted value for no change in therapy. Notably, the prediction gap between no change and change at 6 months is between 0.56 and 0.8 probability of itching development. The effect of “pain development for 6 months” on the “increase in itching” class shows a positive relationship between pain development and the likelihood of experiencing an increase in itching. Finally, the effect of the feature “DLQI development after 6 months” on the “reduction of itching” class suggests that an improvement in QoL is positively predisposing to a reduction in itching.

### Target 2: Pain Development for 6 Months

The feature “pain development for 6 months” consists of 5 different subclasses, with a more even distribution than the previous target subclasses in the training partition. In total, 29.4% and 26.8% were assigned to the subclasses “increase in pain” and “constant low level of pain,” respectively. “Constant severe pain” had the lowest proportion of all feature subclasses, with only 5.1%. The remaining subclasses accounted for 20.4% in the “consistently pain free” and 18.3% in the “reduction of pain” categories.

**Figure 4:**
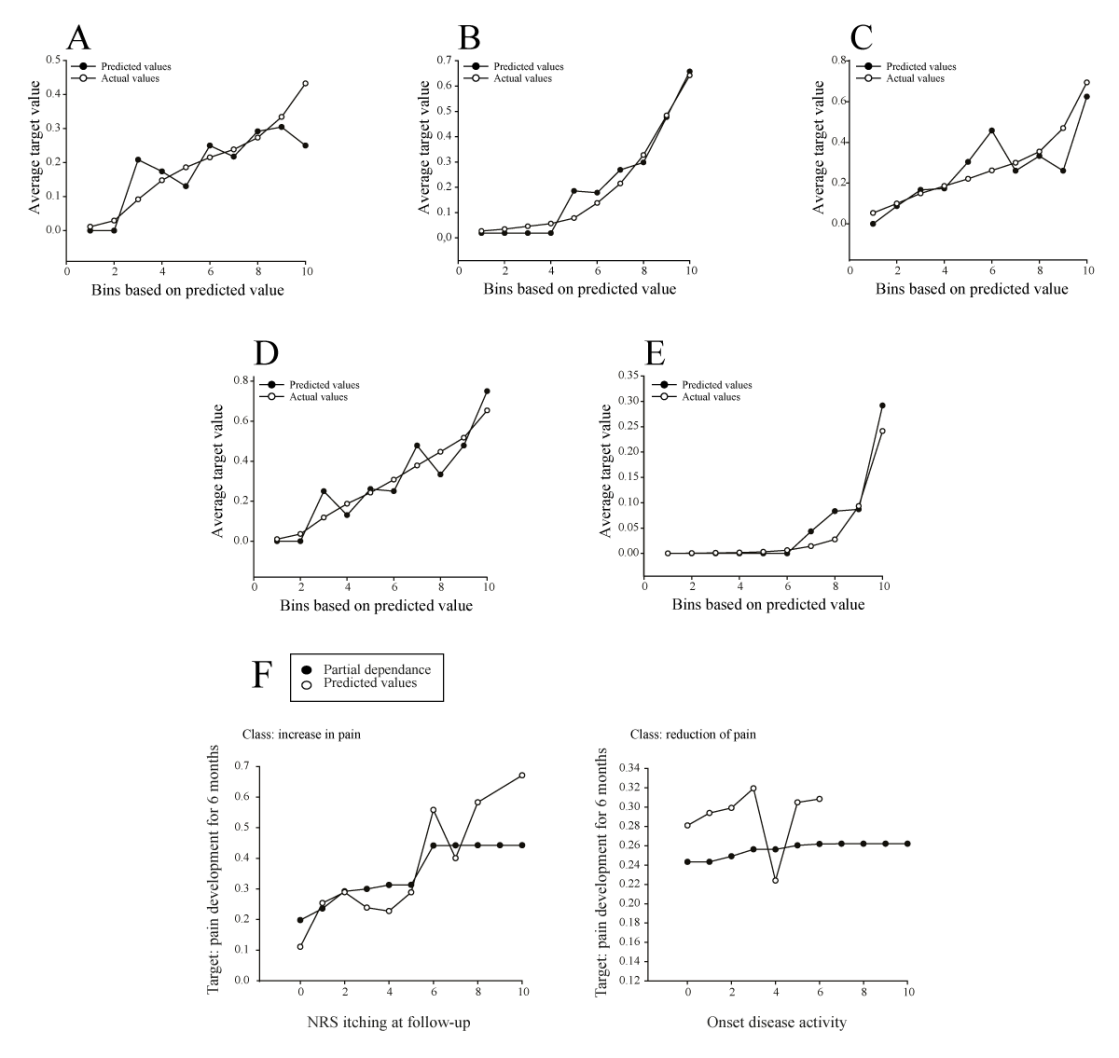
Evaluation of the selected model for the target "pain development for 6 months." Lift charts for all 5 target classes for CV partition: (A) reduction of pain, (B) consistently pain free, (C) constant low level of pain, (D) increase in pain, and (E) constant severe pain. (F) The selection of feature effects of the features "NRS itching at follow-up" on the class "increase in pain" and "onset disease activity" on the class "consistently pain free." All feature effects are shown for the validation partition. CV: cross-validation; DLQI: Dermatology Life Quality Index; NRS: numeric rating scale.

The performance of the selected random forest classifier (Gini) model is shown in the lift charts (Figure 4A-E) and is relatively accurate for most classes, with predicted values closer to the actual values. For the classes “reduction of pain,” “constant low level of pain,” and “increase in pain,” the model performed moderately well with some scatter in the predicted values (Figures 4A, C, and D). For the “consistently pain free” and “constant severe pain” classes, the model performed exceptionally well with solid concordance between actual and predicted values. Nevertheless, all lift charts show an upward trajectory of the curves, indicating that the model is most effective in predicting outcomes in all target classes.

Similar to target 1, the most influential feature for all target classes in this case was “had therapy change” but closely followed by “onset disease activity” with a feature impact of 0.93 (Part B in Multimedia Appendix 8). Notably, “NRS itching at follow-up”(feature impact=0.46) and “NRS itching at onset”(feature impact=0.22) were among the top 5 most impactful aggregated features, confirming that model performance on this target was mostly influenced by itching activity during the study period. A detailed view of how the 3 most influential features affect the target classes is provided in Multimedia Appendix 10. For example, regarding the feature “NRS itching at the 6-month follow-up,” a higher intensity of itching at 6 months may lead to an increase in pain intensity (Figure 4F). This association was observed in both the partial dependence plot and the predicted values. In addition, although the partial dependence curve showed only moderate steepness, a higher level of disease activity at baseline appeared to be more favorable for achieving a reduction in pain status at 6 months, with predicted values further confirming this trend (Figure 4F).

### Target 3: DLQI Development for 6 Months

**Figure 5:**
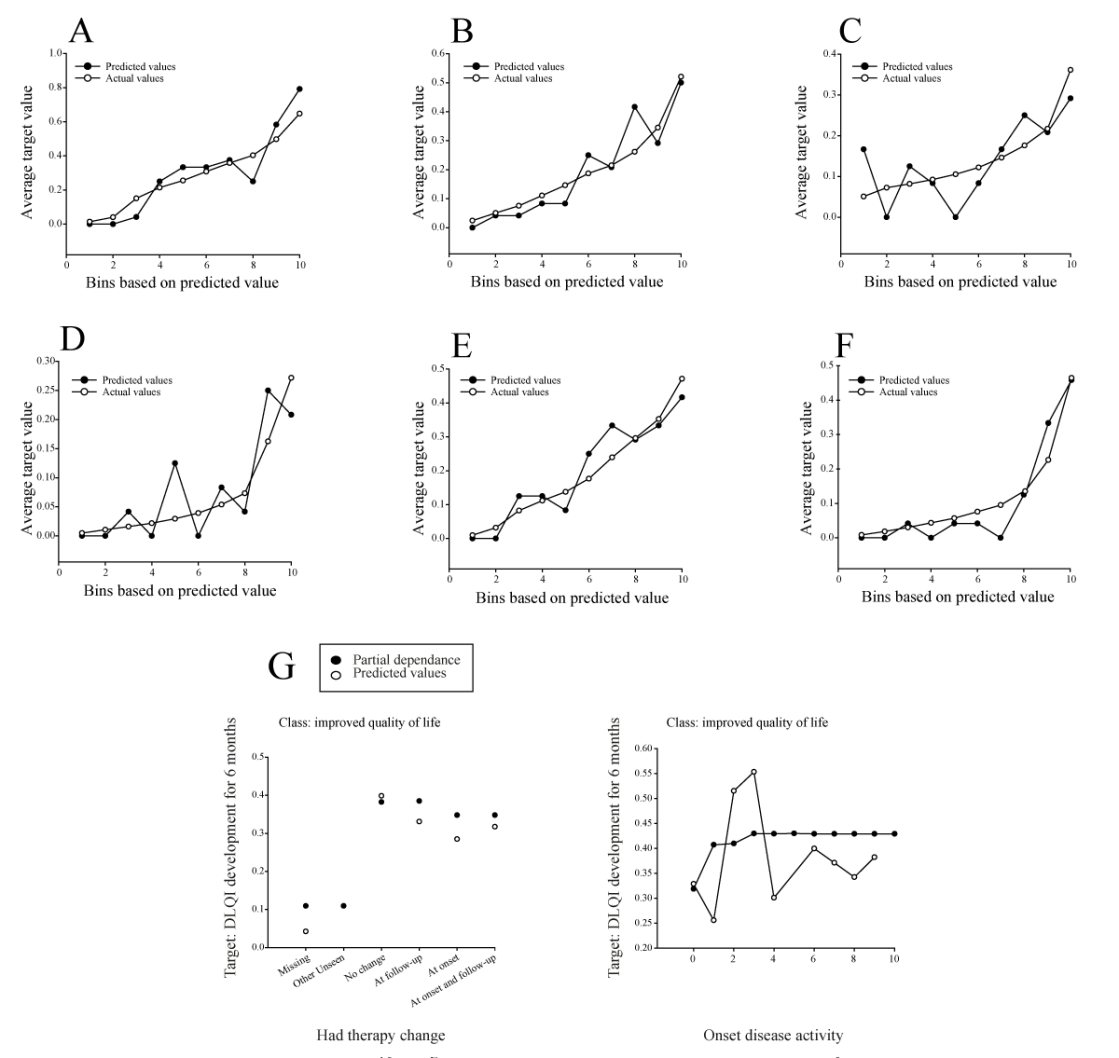
Evaluation of the selected model for the target "DLQI development for 6 months." Lift charts for all 6 target classes for CV partition: (A) improved quality of life, (B) consistently best quality of life, (C) consistently good quality of life, (D) consistently mediocre quality of life, (E) reduction in quality of life, and (F) consistently poor quality of life. (G) The selection of feature effects of the features "had therapy change" on the class "improved quality of life" and "onset disease activity" on the class "consistently best quality of life." All feature effects are shown for the validation partition. CV: cross-validation; DLQI: Dermatology Life Quality Index.

By choosing “DLQI development for 6 months” as a target, we wanted to gain valuable insight into the impact of both the chronic skin conditions analyzed and their treatments on patients’ QoL. The multiclass feature target was also divided into subclasses, including “improved QoL,” “consistently best QoL,” “consistently good QoL,” “consistently mediocre QoL,” “reduction in QoL,” and “consistently poor QoL” (Multimedia Appendix 2). A total of 29.6% (71/240) of the patients in the data sets had an improved QoL, whereas another combined 30% (72/240) either experienced a decline in QoL or had a consistently poor QoL. The remaining 40.4% (97/240) of the patients had an overall favorable QoL throughout the study. During the AutoML analysis, we selected the random forest classifier (Gini) as the optimal model out of the 27 different models trained on the target. The model demonstrated accuracy, as evidenced by the upward trend in the lift charts (Figure 5A-F), with actual and predicted lines aligned relatively close to each other. However, the model performed best in predicting deterioration or improvement in QoL (Figure 5A and E).

Notably, therapy change and disease activity at onset also appear to be the most influential features in calculating DLQI development for 6 months with the chosen model (Part C in Multimedia Appendix 8). In addition, the itching intensity at onset qualifies as the third most impactful feature. Detailed graphical representations of feature effects for all DLQI development subclasses are provided, with the 3 highest-scoring feature effects selected for each subclass (Multimedia Appendix 11). In terms of change in therapy, both no change in therapy and a change in therapy at 6 months showed the strongest positive effect on improvement in QoL (partial dependence=0.38 for both subclasses). Furthermore, when considering partial dependence, improved QoL was associated with higher disease activity at baseline, although the predicted values were somewhat scattered (Figure 5G).

### Target 4: App Use

**Figure 6:**
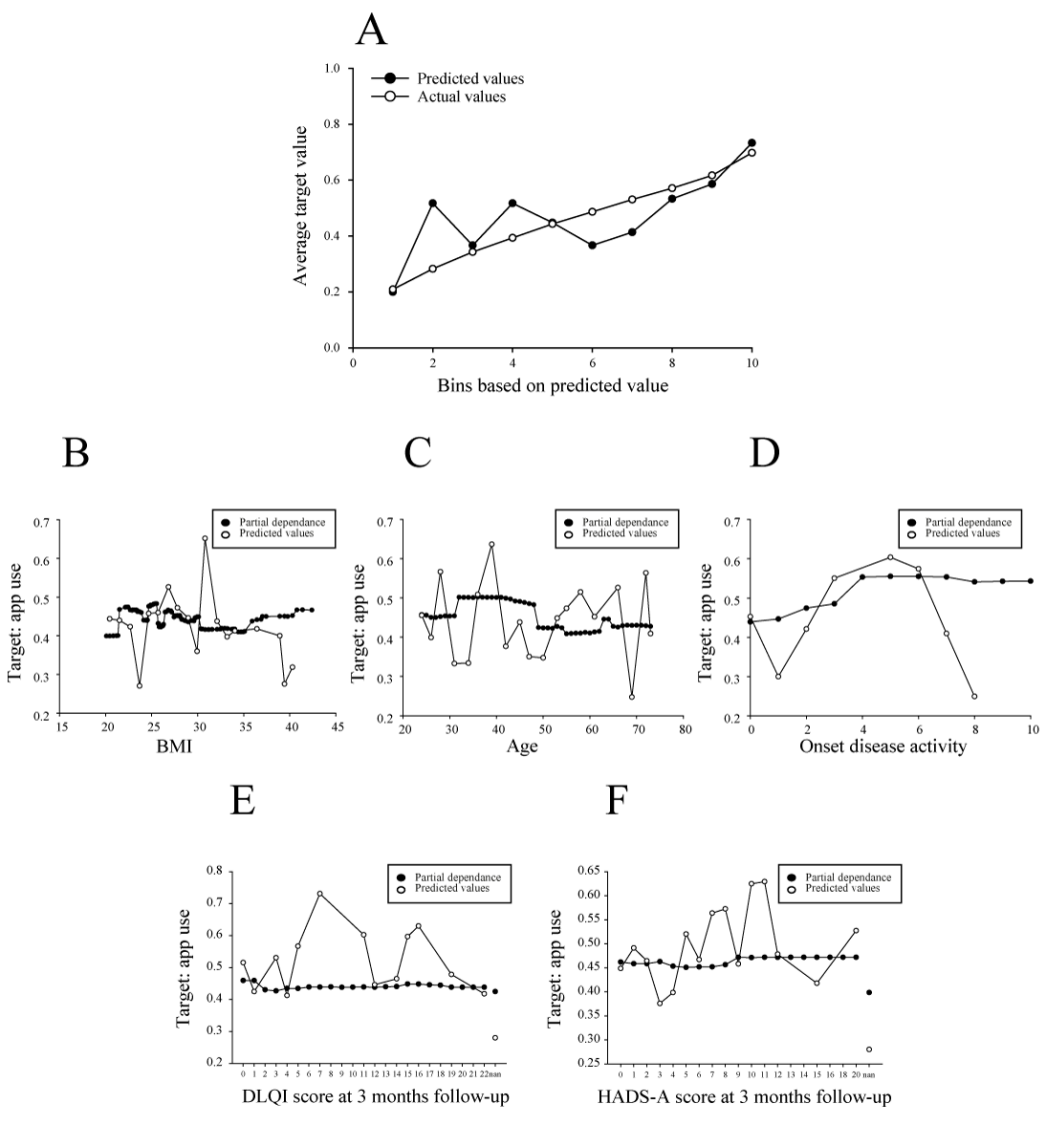
Evaluation of the selected model for the target "app use." (A) Model lift chart for CV partition. The selection of feature effects of the features (B) "BMI," (C) "Age," (D) "onset disease activity," (E) "DLQI score at 3 months follow-up," and (F) "HADS-A at 3 months follow-up." All feature effects are shown for the validation partition. CV: cross-validation; DLQI: Dermatology Life Quality Index; HADS-A: Hospital Anxiety and Depression Scale–Anxiety.

The ENET blender model outperformed all other models trained on the binary target “app use.” in total, 48.1% (142/295) of patients in the secondary training data set used the app at least once. The model lift chart demonstrates accuracy, as seen by the upward trajectory and closeness of the actual and predicted values for the CV partition (Figure 6A). This is further supported by the model’s ROC and lift and gain curves, which show reliable performance in the holdout partition (Multimedia Appendix 12). App use was classified as a binary yes or no feature (Multimedia Appendix 2), with 295 patients in the training partition having used the app (Multimedia Appendix 8). During the AutoML exploratory data analysis, all other app data features were excluded as they were automatically classified as data leakage.

Overall, the most influential features were “BMI,” “age,” and “onset disease activity” (Figure 6B-D; Part D in Multimedia Appendix 8). Accordingly, the feature impact values for these 3 features were 1.00, 0.996, and 0.852, respectively. When considering partial dependence, app use appeared to decrease and plateau between BMIs of 30 and 35 and tended to increase for BMIs >35 (Figure 6B). For the feature “age,” patients >47 years tended to use the app less than their younger counterparts (Figure 6C). Notably, patients aged between 23 and 31 years also seemed to use the app less than middle-aged patients. Notably, the likelihood of using the app increased when disease activity was >2 out of 10, reaching a higher plateau from 4 out of 10 (Figure 6D). Finally, the DLQI score at 3 months had a constant influence on app use (Figure 6E), whereas a HADS-A score >8 seemed to have a slightly better influence on app use, as seen with partial dependence and evidenced by its predicted values (Figure 6F).

## Discussion

### Principal Findings

In the wake of the global digitalization wave, health care systems are undergoing significant changes, with mHealth showing promising improvements in health outcomes and services [48]. However, there is still a need for additional clinical effectiveness studies across a wider range of health care services [49,50]. This study focused on psoriasis and hand and foot eczema, 2 common chronic inflammatory skin conditions, and explored the effect of a smartphone monitoring app on disease activity and symptoms. To be specific, we used AutoML to analyze clinical data sets from patients with dermatologic conditions who used a medical smartphone app during interventional studies at our university hospital from 2018 to 2021. On the basis of the findings of our ML models, various associations of the data sets were discovered in relation to the selected targets of itching, pain, QoL after 6 months of study, and app use. There is evidence that these parameters affect disease manifestation progression and could provide a framework for building predictive models. Even if physicians and researchers do not have extensive expertise in data science, it is crucial to provide user-friendly and intuitive applications of these methods, such as AutoML [51]. We suggest several factors that should be considered in the management of patients with psoriasis and atopic hand and foot eczema.

Itching, also known as pruritus, affects up to 25% of the population at some point in their lives [52]. In the context of chronic skin conditions, itching becomes a chronic and potentially severe problem, often leading to a vicious cycle of skin damage, psychological distress, and a significant reduction in QoL [53]. Chronic itching is one of the main symptoms of atopic dermatitis, occurring at least once a day in up to 91% of patients with atopic dermatitis [54]. Similarly, up to 84% of patients with psoriasis experience itching [55], although in this condition, it is often underrecognized [56]. Therefore, we chose “itching development for 6 months” as an AutoML target and selected the light gradient boosted trees classifier model first to better understand itch progression between follow-ups and also to uncover relationships or interactions of other data set features with the target. Lift charts and feature impact techniques were used in this evaluation. The lift charts showed that the model was best at predicting the classes “consistently itch free” and “increase in itching,” although the “constant low level of itching” class had the largest data distribution. This is not surprising, particularly because in multiclass ML classification, the class with the highest proportion of distributions does not necessarily have the highest performance metric scores or the best lift chart. The performance of a class is determined by several factors, including the quality of the data, the uniqueness of its features compared with other classes, and the specific model used. The model also showed considerable accuracy in predicting patients who would experience a reduction in itching and a constant low level of itching. However, it was only moderately successful in identifying patients who would either transition to an itch-free state or continue to experience severe itching for the 6-month period. The moderate accuracy of the latter 2 classes could be partly explained by the fact that the highest proportion of the distribution in the training partition belonged to patients with consistently low levels of itching, whereas patients with either no itching or extreme itching were fewer in number and represented outliers. However, this disparity in classification is reasonable given that although ongoing treatment of patients with psoriasis and hand and foot eczema is effective in keeping this symptom under control [57], there are small fractions of patients who either achieve complete remission or are resistant to therapy [58]. Although the small size of these outliers is a testament to the progress of modern therapies, ML models do require a large amount of data to make accurate predictions [59]. Nevertheless, classification models are indeed important for medical use cases [60], with gradient boosted tree classifier models that have already been used effectively in various clinical trials [61-63], further validating our model selection.

Feature impact identified “had therapy change” as the most influential feature, followed by the “DLQI score at the 6-month follow-up,” “onset disease activity,” and “age” in fourth place. It is noteworthy that several features, including various pain categorizations, also played an important role in building the selected model. Itch is collaterally controlled by treatment regimens for underlying skin conditions, with several studies even demonstrating a statistically significant reduction in itch with various systemic therapeutic agents in atopic dermatitis [64]. However, there is no evidence in the literature that a change in therapy is associated with the severity of pruritus. To our knowledge, this is the first time a retrospective clinical data analysis study has shown that a modification of therapy affects the development of itching. Notably, although the differences between the partial dependence plots were minor, changes in therapy at baseline and at follow-up were more likely to achieve a constant low level of itching than no change in therapy. This observation could be explained by the fact that changes in therapy are not necessarily because of higher disease activity but also depend on various factors such as tolerability; intensity of side effects; or demographic, psychosocial, and health system variables [65]. Consequently, a timely change in therapy could be beneficial for the patient’s pruritic symptoms. With regard to the other influential features of this selected model, such as DLQI, disease activity, age, or pain, their influence is indeed supported by both literature evidence and clinical experience. We were able to show that an improvement in QoL (DLQI development) positively predicted a reduction in itch. In this regard, not only has a correlation between itch severity and DLQI been demonstrated [66], but a statistical mediation model has also been used to calculate DLQI in atopic dermatitis using characteristics such as itch, disease severity, and treatment with a specific crisaborole ointment [67]. In a cross-sectional study aimed at characterizing dermatosis-associated pruritus in Chinese patients, both the prevalence and severity of pruritus were associated and increased with age [68]. In addition, itching in psoriasis has been shown to cause insomnia, poor work performance, anxiety, depression, and pain in patients, thus severely affecting their QoL [69-71].

In terms of pain, its co-occurrence with pruritus in psoriasis vulgaris and atopic dermatitis is well known and documented, although their pathogenesis in these skin conditions is still not fully understood [72]. Notably, the correlation between pruritus and pain in patients with psoriasis vulgaris or atopic dermatitis has not been explicitly addressed in the current literature. Therefore, the choice of the outcome “pain development for 6 months” was important to uncover what other features of the data set might also have an impact on this symptom. We selected a random forest classifier model, which is a well-recognized ML tool for both classification and regression problems, to predict pain outcomes [73]. The target feature was classified into 5 subclasses, the largest being “increase in pain” and “constant low level of pain,” whereas “constant severe pain” was the smallest. Unexpectedly, the model demonstrated exceptional accuracy for “consistently pain free” and “constant severe pain” subclasses, the latter being the smallest in size in the training partition. In this case, the accuracy of the model may depend on the selected model type and the specific nature of the data set and task. Random forests are meta-estimators that fit a number of decision trees to different subsamples of the data set and average the results to improve the prediction accuracy [74]. Therefore, they can be used on small data sets, provided overfitting is avoided, as is the case with DataRobot’s AutoML platform, which uses various overfitting protection techniques [75].

Considering the importance of the modeling features, “had therapy change” was the most influential feature, closely followed by “onset disease activity.” There are no current studies linking changes in the treatment of underlying conditions and changes in pain severity. In contrast, it has been proposed that the treatment of skin conditions may not be sufficient to reduce pain because of the prolonged healing process and that practitioners should therefore strive to prescribe supplementary analgesics or promote psychological coping strategies [72]. However, several studies have found an association between Psoriasis Area and Severity Index score changes and selected DLQI domain scores in patients with moderate to severe psoriasis [76] or, more broadly, a link between pain symptoms and disease activity [77] or QoL [78]. This suggests that these score modifications can be used to assess therapeutic efficacy, which would include a reduction in pain symptoms. Our ML model demonstrated exactly this assumption, indicating, for example, that a higher level of disease activity at baseline could lead to a decrease in pain status at 6 months, thereby providing useful insight into therapeutic strategy management. Therefore, the selection of relevant features is in line with the well-established premise of personalized medicine that, owing to the complexity of the underlying mechanisms of pain, tailoring treatment plans based on individual patient characteristics is critical for successful therapy [79].

As a widely accepted tool for quantifying the impact of skin disease on patients’ lives [80], we next aimed to examine DLQI development for 6 months in patients from our secondary data set. Considering the different subclasses of DLQI development in the training partition, approximately 29.6% of the participants experienced an improvement in their QoL. A similar proportion experienced a decline or consistently poor QoL, and the remaining 40.8% reported stable and favorable QoL. This large final proportion indicates the improvement and effectiveness of the treatment of patients with chronic skin diseases in our clinic but also reflects the situation in contemporary medicine, considering that, at least in psoriasis, DLQI scores have shown an improvement since the introduction of biologics [81,82]. The reapplication of the random forest classifier model to the analysis of QoL development has proven valuable, demonstrating good predictive performance, particularly in determining deterioration or improvement in QoL. In this case, it was not surprising to find similar features that played an influential role in the previous 2 AutoML targets, itching and pain development, because we had already observed that several constructed features, including DLQI scores, had an important impact on these 2 symptoms, and the literature shows many associations between them and QoL, as described earlier. “Had therapy change,” “onset disease activity,” and NRS itching or pain at different follow-ups were among the top 5 most influential features on the performance of our selected model. Therapy changes or adjustments are known to affect QoL. Effective therapy usually leads to an improvement in QoL, as has been shown in various medical fields such as psychiatry [83], psychotherapy [84], or even orthodontics [85]. In addition, the relationship between clinical response to therapy and changes in health-related QoL has also been demonstrated in patients with psoriasis [86]. Specifically, improving skin lesions following treatment significantly affected the QoL of these patients. With regard to the “onset of disease activity” and why higher disease activity at baseline leads to an improvement in QoL, this has only been shown for systemic lupus erythematosus, in which the management of disease activity was found to have a significant impact on QoL [87]. Therefore, it can be assumed that patients with psoriasis or chronic hand and foot eczema with higher disease activity at baseline, who must have experienced an improvement in treatment, also had a better DLQI score at follow-up.

Notably, however, HADS-D and HADS-A scores at baseline and follow-up and BMI were the next 5 most impactful features. Although weight loss significantly improves comorbidities and QoL [88], correlations between the HADS score and DLQI were only shown for the dermatologic conditions rosacea [89] and androgenetic alopecia [90]. As far as our 2 dermatologic disease groups are concerned, only for atopic eczema has a study been carried out in which several scores, including the DLQI and HADS, were used to assess patients’ QoL and levels of depression and anxiety. The study did not explicitly mention a correlation between the DLQI and HADS scores but concluded that atopic eczema was associated with depression and anxiety [91]. Finally, regarding the HADS score and psoriasis, it is worth noting that our working group has recently shown that in a clinical trial of patients with psoriasis included in this secondary data set, a significant improvement in both HADS-A and HADS-D scores was observed in those patients who used the monitoring app [15,16].

Knowing that our disease monitoring smartphone app had a positive effect on the mental health of patients with psoriasis, we were interested in determining how the disease scores or patient features of our data set influenced their use of the app. For this purpose, we selected an ENET ensemble or blender model to predict the use of the monitoring medical app among our patients with chronic hand and foot eczema and psoriasis. We found that this model outperformed all the other 216 models trained on binary data, indicating whether a patient used the app or not. ENET is a regression method that combines 2 of the most commonly used regularized linear regression techniques: lasso and ridge [92]. It is suitable for cases in which there are several correlated features, as it favors the selection of groups of correlated variables. For example, ENET regression was used to investigate the association of sociodemographic factors with COVID-19 case rates [93]. In addition, a stacking ensemble learning framework incorporating ENET was used to predict genomic estimated breeding values [94].

The most important determinants of app use, as predicted by the model, were “BMI,” “age,” and “onset disease activity,” in descending order of influence. Patients with a BMI <30 and >35, aged between 31 and 47 years, and with a disease activity score >2 out of 10 were more likely to use the app. Although some studies have examined the use of apps specifically designed for obesity management, there are not many publications describing smartphone use and its association with BMI. Notably, an association between smartphone use for entertainment and obesity has only been shown in school-aged children and adolescents [95]. This partly reflects our observation that app use was more likely in patients with BMI >35. In terms of age, it is generally known that older age groups are less likely to use mobile phone apps. A study investigating the factors influencing the low use of mHealth apps by people aged ≥50 years concluded that almost half of the study cohort lacked adequate knowledge of mobile technology [96]. For younger patients, another trial found that adults with a mean age of around 24 years expressed willingness to use apps for behavior change but placed a high value on the accuracy, legitimacy, and safety of the app [97]. Therefore, health care professionals may want to consider factors in these age groups when using medical apps. Several studies have shown associations and correlations between disease activity and the use of medical apps. However, the nature and extent of these associations appear to vary depending on the type of disease and the specific features and capabilities of the app. A population-based survey published in 2019 showed that among patients with cardiovascular disease and diabetes mellitus, almost 25% used smartphone apps for health-related purposes [98]. However, their primary goals were not to monitor diseases but mostly to improve them through physical activity or weight loss. Another study found an association between the use of smartphone self-management apps and medication adherence in patients with asthma and chronic obstructive pulmonary disease [99]. Given the suboptimal levels of adherence to controller medications for these conditions, apps have the potential to improve patient outcomes and potentially reduce health care costs.

It is also noteworthy that the DLQI score at 3 months had a constant influence on app use. In other words, worsening or improving QoL did not seem to change app use. This is also reflected in the observation that in our target “DLQI development for 6 months,” app use did not appear among the most influential target features. In addition, there are studies that could not show a significant change in DLQI in patients using a medical app [100]. In contrast, telemedicine as a remote clinical service is known to improve the DLQI score [101,102]. Our work group has shown that in patients with hand and foot eczema, an app use frequency of less than once every 5 weeks had a significant improvement in the DLQI score [47]. Considering the reports in the current literature of low mHealth app influences on DLQI changes and the fact that we used a combined data set of patients with psoriasis and eczema, it is understandable that DLQI is a key determinant of app use, but its variations do not affect our chosen ML target. Finally, our research group has previously shown that the use of the monitoring app led to a significant reduction in HADS-D scores in patients with psoriasis. This was the case for patients who used the app less than once a month [15]. A reduction in both HADS-A and HADS-D scores was also observed when the app was used less than once every 5 weeks [16]. Although this prior influence was not observed in our hand and foot eczema study [47], it was interesting to observe that a borderline HADS-A score starting at 8 did indeed seem to have at least a slight influence on app use. It could, therefore, be argued that patients with anxiety symptoms may be more likely to use health monitoring apps. This is important, as it has been shown in patients with psoriasis that a cloud-based interactive management program led to a reduction in anxiety [102], whereas a self-help app specifically designed to treat depression and anxiety symptoms led to a significant reduction in these symptoms [103]. In conclusion, app use was significantly influenced by a combination of BMI, age, and disease activity, among other variables. This suggests that personalized interventions that take these factors into account may increase app use and potentially improve patient outcomes. Future studies could investigate the causal mechanisms driving these associations and test interventions to promote app use.

### Conclusions

Our findings pave the way for further research into the clinical application of various ML techniques to assist patients with dermatologic conditions with diagnosis, therapy, and communication outside of appointments using medical smartphone apps. However, the results of ML analysis should be interpreted with caution and in the context of existing research, as the representativeness of such an ML model can be challenging [104]. It is worth noting that most of the feature effects in this study were small. Although the selected ML models had relatively high metric scores, the minor differences in feature effects might be because of the size of the secondary data set. The generalizability of the selected ML models should be considered carefully, as the patient pool was small compared with other ML studies. More patients may have increased the number of observed differences. In addition, the inclusion of laboratory diagnostics, which were not incorporated in the secondary data set because of high heterogeneity, might have provided further insights. Therefore, future research should address this issue by developing more sophisticated ML models using more diverse data sets. Despite these limitations, this study provides a framework upon which predictive models can be built. We demonstrated the effectiveness of applying ML to existing data sets to discover new relationships that could not be uncovered using traditional regression analysis. In addition, we support several elements that should be considered in our patients’ treatment regimens. Chronic inflammatory skin diseases cause numerous individual, societal, and economic problems; however, their clinical management is not fully optimized to meet patient expectations in a therapeutic environment. Future prospective research will provide the necessary context to determine how effective the combination of ML prediction and medical smartphone use is in real-world practice and whether their implementation has the potential to be registered as a medical product.

## Appendix

Multimedia Appendix 1 Graphical layout of the calculation of selected new features. Calculation details are documented in Multimedia Appendix 2.

Multimedia Appendix 2 Creation of new features for the secondary data sets. To enrich our existing data set, several new features were created from the existing data sets, including categorical features over study trial time. This was done by combining onset and follow-up (at 0 and 6 months) data points, as seen in the calculation method column for variables containing "development." A feature with multiple data categories is defined as a multiclass feature. Smartphone app log data were also exported and added to the data set to facilitate app use data and question results.

Multimedia Appendix 3 Machine learning processing details and summary statistics. For each target, the features used for modeling and summary statistics are given in "Features used for modeling and summary statistics." Metrics such as mean, median and SD are given, as well as the type of parameter (numeric and categorical). The number of unique values, minimum and maximum values are also provided. Finally, a target leakage assessment is performed, classifying characteristics as low, medium or high risk for target leakage. The "Data Quality Handling Report" covers the handling of categorical features, missing value imputation and optimization parameters. For target 4, imputation techniques are not provided as the Blender model cannot summarize this information in a single table for all models. For each cross-validation fold, the primary modeling performance metric is documented in the "Cross-Validation Scores.".

Multimedia Appendix 4 List of features manually and automatically extracted from the primary data sets by the investigators and the AutoML platform to be used in the machine learning process. For the features "itching development for 6 months,” "pain development for 6 months,” and "DLQI development for 6 months,” we excluded the 3-month follow-up data as the data points were sparse. However, for the target "app use,” we did not exclude these data points as app monitoring was more frequent than clinical visits and therefore less prone to biased during AutoML analysis. For the app use target, a Blender model was created from the top 3 models in the platform ranking. The AutoML platform automatically performs ML model development on the initially selected feature list, the "Regular Feature List," but also revises the process with a second reduced feature list. Unless otherwise stated, "at follow-up" means 6 months after study entry. AutoML: automated machine learning; ML: machine learning.

Multimedia Appendix 5 Graphical representation of data partitioning performed by the automated machine learning (AutoML) platform.

Multimedia Appendix 6 Selected model performance assessments shown for validation, CV, and holdout partitions. LogLoss score and AUC were considered as the main model metrics. The platform was instructed to model for the highest LogLoss score, indicating the model's relationship between actual and predicted values. Lower LogLoss scores indicate closer actual and predicted values. AUC indicates the ability to discriminate between feature classes (ie, yes or no). FVE multinomial (binomial for binary yes-or-no features applies only for the app use target) measures the deviation between the specific model and a "perfect model," which explains 100% of the variability in the data. AUC: area under the curve; CV: cross-validation; FVE: fraction of variance explained.

Multimedia Appendix 7 Learning curves for performance measures. Learning curves illustrate how the performance of individual models varies as the sample size changes. The average metric values (LogLoss, AUC, and accuracy for targets 1-3; and LogLoss, AUC, and Mathews correlation coefficient for target 4) are shown with a 95% CI. During the training process of these machine learning models, the data sample size is subsequently increased for models that perform well. The performance metric is then evaluated in relation to the training sample size and is reported here as the average metric, with the 95% CI indicating the range during the training process. The sample sizes were: 16, 32, 64 and 80%. Models with the same machine learning blueprints are grouped into one average model in this representation. AUC: area under the curve.

Multimedia Appendix 8 Feature impact showing the top 10 features with the greatest impact on model performance, listed in descending order of normalized impact for all target classes aggregated. The graph is shown for the target (A) "itching development for 6 months," (B) "pain development for 6 months," (C) "DLQI development for 6 months," and (D) "app use." Also, data distribution for the target "app use" is depicted (E). DLQI: Dermatology Life Quality Index.

Multimedia Appendix 9 Feature effects of the 3 most impactful features for each target class of "itching development for 6 months": (A) reduction of itching, (B) consistently itch free, (C) became free of itching, (D) constant low level of itching, (E) increase in itching, and (F) constant severe itching. For each class, we present the first 3 feature effects according to the class's own feature impact ranking. Each feature effect shows a partial dependence plot, which shows the effect of a feature when its values are changed and all other features in the model remain unchanged. This allows for a better interpretation of the results and may show nonlinear associations between the target feature and a second specific feature. Predicted features are also presented in the graphs.

Multimedia Appendix 10 Feature effects of the 3 most impactful features for each target class of "pain development for 6 months": (A) reduction of pain, (B) consistently pain free, (C) constant low level of pain, (D) increase in pain, and (E) constant severe pain. For each class, we present the first 3 feature effects according to the class's own feature impact ranking. Each feature effect shows a partial dependence plot, which shows the effect of a feature when its values are changed and all other features in the model remain unchanged. This allows for a better interpretation of the results and may show nonlinear associations between the target feature and a second specific feature. Predicted features are also presented in the graphs.

Multimedia Appendix 11 Feature effects of the 3 most impactful features for each target class of "DLQI development for 6 months": (A) improved quality of life, (B) consistently best quality of life, (C) consistently good quality of life, (D) consistently mediocre quality of life, (E) reduction in quality of life, and (F) consistently poor quality of life. For each class, we present the first 3 feature effects according to the class's own feature impact ranking. Each feature effect shows a partial dependence plot, which shows the effect of a feature when its values are changed and all other features in the model remain unchanged. This allows for a better interpretation of the results and may show nonlinear associations between the target feature and a second specific feature. Predicted features are also presented in the graphs.

Multimedia Appendix 12 Performance charts for the target "app use." (A) The ROC curve shows the false positive rates (x-axis) against the true positive rates (y-axis). A perfect model would fit the curve in the upper right corner of the graph. (B) The lift curve shows the lift of the model compared to the threshold calculated by the model. (C) The gain chart depicts sensitivity and specificity values for different percentages of the model predictions. All plots are shown for the holdout partition. ROC: receiver operating characteristic.

## Abbreviations

AUC: area under the curve
AutoML: automated machine learning
CV: cross-validation
DLQI: Dermatology Life Quality Index
ENET: elastic net
FVE: fraction of variance explained
HADS: Hospital Anxiety and Depression Scale
HADS-A: Hospital Anxiety and Depression Scale-Anxiety
HADS-D: Hospital Anxiety and Depression Scale-Depression
ICD-10: International Classification of Diseases, Tenth Revision
mHealth: mobile health
ML: machine learning
NRS: numeric rating scale
QoL: quality of life
ROC: receiver operating characteristic

## Data Availability

The pseudonymized secondary data set containing only common and new calculated features used for AutoML is deposited on the web [105].

## References

[1] Amankwah-Amoah J, Khan Z, Wood G, Knight G (2021). COVID-19 and digitalization: the great acceleration. J Bus Res.

[2] Tuckson RV, Edmunds M, Hodgkins ML (2017). Telehealth. N Engl J Med.

[3] Meystre S (2005). The current state of telemonitoring: a comment on the literature. Telemed J E Health.

[4] Barkai G, Gadot M, Amir H, Menashe M, Shvimer-Rothschild L, Zimlichman E (2021). Patient and clinician experience with a rapidly implemented large-scale video consultation program during COVID-19. Int J Qual Health Care.

[5] Clark B (2022). Cellular Phones as a primary communications device: what are the implications for a global community?. Glob Media J.

[6] Marshall C, Lewis D, Whittaker M (2013). Strengthening health systems in mHealth technologies in developing countries: a feasibility assessment and a proposed framework 2013. Health Information Systems Knowledge Hub School of Populaiton Health University of Queensland.

[7] Coleman J, Bohlin KC, Thorson A, Black V, Mechael P, Mangxaba J, Eriksen J (2017). Effectiveness of an SMS-based maternal mHealth intervention to improve clinical outcomes of HIV-positive pregnant women. AIDS Care.

[8] Ming LC, Hameed MA, Lee DD, Apidi NA, Lai PS, Hadi MA, Al-Worafi YM, Khan TM (2016). Use of medical mobile applications among hospital pharmacists in Malaysia. Ther Innov Regul Sci.

[9] Hall CS, Fottrell E, Wilkinson S, Byass P (2014). Assessing the impact of mHealth interventions in low- and middle-income countries--what has been shown to work?. Glob Health Action.

[10] Sauermann S, Herzberg J, Burkert S, Habetha S (2021). DiGA - a chance for the German healthcare system. J Eur CME.

[11] Gerke S, Stern AD, Minssen T (2020). Germany's digital health reforms in the COVID-19 era: lessons and opportunities for other countries. NPJ Digit Med.

[12] de Santis KK, Jahnel T, Sina E, Wienert J, Zeeb H (2021). Digitization and health in Germany: cross-sectional nationwide survey. JMIR Public Health Surveill.

[13] Weitzel EC, Quittschalle J, Welzel FD, Löbner M, Hauth I, Riedel-Heller SG (2021). [E-Mental Health and healthcare apps in Germany]. Nervenarzt.

[14] Donker T, Petrie K, Proudfoot J, Clarke J, Birch MR, Christensen H (2013). Smartphones for smarter delivery of mental health programs: a systematic review. J Med Internet Res.

[15] Beck A, Schulze-Hagen T, Domogalla L, Herr R, Benecke J, Schmieder A (2021). Effect of a disease-monitoring smartphone application in combination with a patient educational program on mental health of patients with psoriasis: a randomized intervention study. J Am Acad Dermatol.

[16] Domogalla L, Beck A, Schulze-Hagen T, Herr R, Benecke J, Schmieder A (2021). Impact of an eHealth smartphone app on the mental health of patients with psoriasis: prospective randomized controlled intervention study. JMIR Mhealth Uhealth.

[17] Bubak C, Schaarschmidt ML, Schöben L, Peitsch WK, Schmieder A (2019). Analyzing the value of an educational program for psoriasis patients: a prospective controlled pilot study. BMC Public Health.

[18] Daré LO, Bruand PE, Gérard D, Marin B, Lameyre V, Boumédiène F, Preux PM (2019). Co-morbidities of mental disorders and chronic physical diseases in developing and emerging countries: a meta-analysis. BMC Public Health.

[19] Oliveira MdFSPd, Rocha BdO, Duarte GV (2015). Psoriasis: classical and emerging comorbidities. An Bras Dermatol.

[20] Mease PJ, Gladman DD, Papp KA, Khraishi MM, Thaçi D, Behrens F, Northington R, Fuiman J, Bananis E, Boggs R, Alvarez D (2013). Prevalence of rheumatologist-diagnosed psoriatic arthritis in patients with psoriasis in European/North American dermatology clinics. J Am Acad Dermatol.

[21] Boehncke W, Schön MP (2015). Psoriasis. Lancet.

[22] Rapp SR, Feldman SR, Exum ML, Fleischer AB Jr, Reboussin DM (1999). Psoriasis causes as much disability as other major medical diseases. J Am Acad Dermatol.

[23] Sanchez-Carazo JL, López-Estebaranz JL, Guisado C (2014). Comorbidities and health-related quality of life in Spanish patients with moderate to severe psoriasis: a cross-sectional study (Arizona study). J Dermatol.

[24] Conlon EG, Wright KT (2019). A comparison of two chronic skin conditions: atopic dermatitis and psoriasis. J Spec Oper Med.

[25] Chen WY, Chen SC, Hsu SY, Lin YA, Shih CM, Huang CY, Wang KH, Lee AW (2022). Annoying psoriasis and atopic dermatitis: a narrative review. Int J Mol Sci.

[26] Newsom M, Bashyam AM, Balogh EA, Feldman SR, Strowd LC (2020). New and emerging systemic treatments for atopic dermatitis. Drugs.

[27] Psomadakis CE, Han G (2019). New and emerging topical therapies for psoriasis and atopic dermatitis. J Clin Aesthet Dermatol.

[28] Wang J, Wang Y, Wei C, Yao NA, Yuan A, Shan Y, Yuan C (2014). Smartphone interventions for long-term health management of chronic diseases: an integrative review. Telemed J E Health.

[29] Howard J (2019). Artificial intelligence: implications for the future of work. Am J Ind Med.

[30] Habehh H, Gohel S (2021). Machine learning in healthcare. Curr Genomics.

[31] Shilo S, Rossman H, Segal E (2020). Axes of a revolution: challenges and promises of big data in healthcare. Nat Med.

[32] Waring J, Lindvall C, Umeton R (2020). Automated machine learning: Review of the state-of-the-art and opportunities for healthcare. Artif Intell Med.

[33] - (2020). Big hopes for big data. Nat Med.

[34] Esteva A, Robicquet A, Ramsundar B, Kuleshov V, DePristo M, Chou K, Cui C, Corrado G, Thrun S, Dean J (2019). A guide to deep learning in healthcare. Nat Med.

[35] Benecke J, Benecke C, Ciutan M, Dosius M, Vladescu C, Olsavszky V (2021). Retrospective analysis and time series forecasting with automated machine learning of ascariasis, enterobiasis and cystic echinococcosis in Romania. PLoS Negl Trop Dis.

[36] Olsavszky V, Dosius M, Vladescu C, Benecke J (2020). Time series analysis and forecasting with automated machine learning on a national ICD-10 database. Int J Environ Res Public Health.

[37] Chan S, Reddy V, Myers B, Thibodeaux Q, Brownstone N, Liao W (2020). Machine learning in dermatology: current applications, opportunities, and limitations. Dermatol Ther (Heidelb).

[38] Das K, Cockerell CJ, Patil A, Pietkiewicz P, Giulini M, Grabbe S, Goldust M (2021). Machine learning and its application in skin cancer. Int J Environ Res Public Health.

[39] Géraud C, Griewank KG (2020). Re: Deep learning outperformed 11 pathologists in the classification of histopathological melanoma images. Eur J Cancer.

[40] Yu K, Syed MN, Bernardis E, Gelfand JM (2020). Machine learning applications in the evaluation and management of psoriasis: a systematic review. J Psoriasis Psoriatic Arthritis.

[41] De A, Sarda A, Gupta S, Das S (2020). Use of artificial intelligence in dermatology. Indian J Dermatol.

[42] Chou A, Torres-Espin A, Kyritsis N, Huie JR, Khatry S, Funk J, Hay J, Lofgreen A, Shah R, McCann C, Pascual LU, Amorim E, Weinstein PR, Manley GT, Dhall SS, Pan JZ, Bresnahan JC, Beattie MS, Whetstone WD, Ferguson AR, TRACK-SCI Investigators (2022). Expert-augmented automated machine learning optimizes hemodynamic predictors of spinal cord injury outcome. PLoS One.

[43] Marmolejo-Ramos F, Vélez JI, Romão X (2015). Automatic detection of discordant outliers via the Ueda’s method. J Stat Distrib App.

[44] Bonannella C, Hengl T, Heisig J, Parente L, Wright MN, Herold M, de Bruin S (2022). Forest tree species distribution for Europe 2000-2020: mapping potential and realized distributions using spatiotemporal machine learning. PeerJ.

[45] Ferri C, Hernández-Orallo J, Modroiu R (2009). An experimental comparison of performance measures for classification. Pattern Recognit Lett.

[46] Manzali Y, Chahhou M, Mohajir ME (2017). Impure decision trees for Auc and log loss optimization. Proceedings of the 017 International Conference on Wireless Technologies, Embedded and Intelligent Systems.

[47] Weigandt WA, Schardt Y, Bruch A, Herr R, Goebeler M, Benecke J, Schmieder A (2023). Impact of an eHealth smartphone app on quality of life and clinical outcome of patients with hand and foot eczema: prospective randomized controlled intervention study. JMIR Mhealth Uhealth.

[48] Paglialonga A, Patel AA, Pinto E, Mugambi D, Keshavjee K, Andreoni G, Perego P, Frumento E (2019). The healthcare system perspective in mHealth. m_Health Current and Future Applications.

[49] Choi W, Wang S, Lee Y, Oh H, Zheng Z (2020). A systematic review of mobile health technologies to support self-management of concurrent diabetes and hypertension. J Am Med Inform Assoc.

[50] Snoswell CL, Chelberg G, de Guzman KR, Haydon HH, Thomas EE, Caffery LJ, Smith AC (2023). The clinical effectiveness of telehealth: a systematic review of meta-analyses from 2010 to 2019. J Telemed Telecare.

[51] Quer G, Arnaout R, Henne M, Arnaout R (2021). Machine learning and the future of cardiovascular care: JACC state-of-the-art review. J Am Coll Cardiol.

[52] Matterne U, Apfelbacher CJ, Vogelgsang L, Loerbroks A, Weisshaar E (2013). Incidence and determinants of chronic pruritus: a population-based cohort study. Acta Derm Venereol.

[53] Capec S, Petrek M, Capec G, Yaremkevych R, Andrashko Y (2022). Psychologic interventions in patients with the chronic dermatologic itch in atopic dermatitis and psoriasis: a step forward with family constellations seminars. Front Med (Lausanne).

[54] Dawn A, Papoiu AD, Chan YH, Rapp SR, Rassette N, Yosipovitch G (2009). Itch characteristics in atopic dermatitis: results of a web-based questionnaire. Br J Dermatol.

[55] Prignano F, Ricceri F, Pescitelli L, Lotti T (2009). Itch in psoriasis: epidemiology, clinical aspects and treatment options. Clin Cosmet Investig Dermatol.

[56] Elewski B, Alexis AF, Lebwohl M, Stein Gold L, Pariser D, Del Rosso J, Yosipovitch G (2019). Itch: an under-recognized problem in psoriasis. J Eur Acad Dermatol Venereol.

[57] Hong J, Buddenkotte J, Berger TG, Steinhoff M (2011). Management of itch in atopic dermatitis. Semin Cutan Med Surg.

[58] Legat FJ (2021). Itch in atopic dermatitis - what is new?. Front Med (Lausanne).

[59] Alqaissi EY, Alotaibi FS, Ramzan MS (2022). Modern machine-learning predictive models for diagnosing infectious diseases. Comput Math Methods Med.

[60] Moreno-Ibarra MA, Villuendas-Rey Y, Lytras MD, Yáñez-Márquez C, Salgado-Ramírez JC (2021). Classification of diseases using machine learning algorithms: a comparative study. Mathematics.

[61] Oguz BU, Shinohara RT, Yushkevich PA, Oguz I (2017). Gradient boosted trees for corrective learning. Mach Learn Med Imaging.

[62] Seto H, Oyama A, Kitora S, Toki H, Yamamoto R, Kotoku J, Haga A, Shinzawa M, Yamakawa M, Fukui S, Moriyama T (2022). Gradient boosting decision tree becomes more reliable than logistic regression in predicting probability for diabetes with big data. Sci Rep.

[63] Zhang Z, Zhao Y, Canes A, Steinberg D, Lyashevska O, written on behalf of AME Big-Data Clinical Trial Collaborative Group (2019). Predictive analytics with gradient boosting in clinical medicine. Ann Transl Med.

[64] Tan XL, Thomas BR, Tan YJ, O'Toole EA (2022). Effects of systemic therapies on pruritus in adults with atopic dermatitis: a systematic review and meta-analysis. Clin Exp Dermatol.

[65] Sokka T, Mäkinen H (2006). Improving outcomes in rheumatoid arthritis: what determines decisions to change ineffective therapy?. J Rheumatol.

[66] Jaworecka K, Rzepko M, Marek-Józefowicz L, Tamer F, Stefaniak AA, Szczegielniak M, Chojnacka-Purpurowicz J, Gulekon A, Szepietowski JC, Narbutt J, Owczarczyk-Saczonek A, Reich A (2022). The impact of pruritus on the quality of life and sleep disturbances in patients suffering from different clinical variants of psoriasis. J Clin Med.

[67] Simpson EL, Tom WL, Bushmakin AG, Cappelleri JC, Yosipovitch G, Ständer S, Luger T, Sanders P, Gerber RA, Myers DE (2021). Relationship among treatment, pruritus, investigator's static global assessment, and quality of life in patients with atopic dermatitis. Dermatol Ther (Heidelb).

[68] Wang X, Lai Q, Zheng B, Ye L, Wen S, Yan Y, Yang B, Man MQ (2021). Both prevalence and severity of pruritus are associated with age in Chinese patients with skin diseases. Clin Cosmet Investig Dermatol.

[69] Amatya B, Wennersten G, Nordlind K (2008). Patients' perspective of pruritus in chronic plaque psoriasis: a questionnaire-based study. J Eur Acad Dermatol Venereol.

[70] Yosipovitch G, Goon A, Wee J, Chan YH, Goh CL (2000). The prevalence and clinical characteristics of pruritus among patients with extensive psoriasis. Br J Dermatol.

[71] Zachariae R, Zachariae CO, Lei U, Pedersen AF (2008). Affective and sensory dimensions of pruritus severity: associations with psychological symptoms and quality of life in psoriasis patients. Acta Derm Venereol.

[72] Pojawa-Gołąb M, Reich A (2020). Skin pain in patients with atopic dermatitis or psoriasis: a web-based survey. Acta Derm Venereol.

[73] Svetnik V, Liaw A, Tong C, Culberson JC, Sheridan RP, Feuston BP (2003). Random forest: a classification and regression tool for compound classification and QSAR modeling. J Chem Inf Comput Sci.

[74] Pedregosa F, Varoquaux G, Gramfort A, Michel V, Thirion B, Grisel O, Blondel M, Prettenhofer P, Weiss R, Dubourg V, Vanderplas J, Passos A, Cournapeau D (2011). Scikit-learn: machine learning in Python. J Mach Learn Res.

[75] Overfitting. DataRobot.

[76] Houghton K, Patil D, Gomez B, Feldman SR (2021). Correlation between change in psoriasis area and severity index and dermatology life quality index in patients with psoriasis: pooled analysis from four phase 3 clinical trials of secukinumab. Dermatol Ther (Heidelb).

[77] Ibrahim F, Ma M, Scott DL, Scott IC (2022). Defining the relationship between pain intensity and disease activity in patients with rheumatoid arthritis: a secondary analysis of six studies. Arthritis Res Ther.

[78] Ogawa K, Fujikoshi S, Montgomery W, Alev L (2015). Correlation between pain response and improvements in patient-reported outcomes and health-related quality of life in duloxetine-treated patients with diabetic peripheral neuropathic pain. Neuropsychiatr Dis Treat.

[79] Rekatsina M, Paladini A, Piroli A, Zis P, Pergolizzi JV, Varrassi G (2020). Pathophysiologic approach to pain therapy for complex pain entities: a narrative review. Pain Ther.

[80] Basra MK, Fenech R, Gatt RM, Salek MS, Finlay AY (2008). The Dermatology Life Quality Index 1994-2007: a comprehensive review of validation data and clinical results. Br J Dermatol.

[81] Armstrong AW, Reich K, Foley P, Han C, Song M, Shen YK, You Y, Papp KA (2019). Improvement in patient-reported outcomes (dermatology life quality index and the psoriasis symptoms and signs diary) with guselkumab in moderate-to-severe plaque psoriasis: results from the phase III VOYAGE 1 and VOYAGE 2 studies. Am J Clin Dermatol.

[82] de Ruiter CC, Rustemeyer T (2022). Biologics can significantly improve Dermatology Life Quality Index (DLQI) in psoriatic patients: a systematic review. Psoriasis (Auckl).

[83] Montgomery W, Kadziola Z, Ye W, Xue HB, Liu L, Treuer T (2015). Correlation between changes in quality of life and symptomatic improvement in Chinese patients switched from typical antipsychotics to olanzapine. Neuropsychiatr Dis Treat.

[84] Crits-Christoph P, Connolly Gibbons MB, Ring-Kurtz S, Gallop R, Stirman S, Present J, Temes C, Goldstein L (2008). Changes in positive quality of life over the course of psychotherapy. Psychotherapy (Chic).

[85] Wang J, Tang X, Shen Y, Shang G, Fang L, Wang R, Xu Y (2015). The correlations between health-related quality of life changes and pain and anxiety in orthodontic patients in the initial stage of treatment. Biomed Res Int.

[86] Lee YW, Park EJ, Kwon IH, Kim KH, Kim KJ (2010). Impact of psoriasis on quality of life: relationship between clinical response to therapy and change in health-related quality of life. Ann Dermatol.

[87] Grau García E, Fragío Gil JJ, Ivorra Cortes J, Ortiz Sanjuan FM, Chalmeta Verdejo I, Román Ivorra JA (2023). The impact of disease activity on health-related quality of life in patients with systemic lupus erythematosus. Med Clin (Barc).

[88] Kolotkin RL, Meter K, Williams GR (2001). Quality of life and obesity. Obes Rev.

[89] Wu Y, Fu C, Zhang W, Li C, Zhang J (2018). The dermatology life quality index (DLQI) and the hospital anxiety and depression (HADS) in Chinese rosacea patients. Psychol Health Med.

[90] Yu L, Moorthy SK, Peng L, Shen L, Han Y, Zhang Z, Li Y, Huang X (2023). Evaluation of anxiety and depression in patients with androgenetic alopecia in shanghai: a cross-sectional study. Dermatol Ther.

[91] Kage P, Poblotzki L, Zeynalova S, Zarnowski J, Simon JC, Treudler R (2022). Depression, anxiety, and suicidal ideation in patients with atopic eczema in a prospective study in Leipzig, Germany. Int Arch Allergy Immunol.

[92] Zou H, Hastie T (2005). Regularization and variable selection via the elastic net. J Royal Statistical Soc B.

[93] Moxley TA, Johnson-Leung J, Seamon E, Williams C, Ridenhour BJ Application of elastic net regression for modeling COVID-19 sociodemographic risk factors. medRxiv. Preprint posted online on January 20, 2023.

[94] Liang M, Chang T, An B, Duan X, Du L, Wang X, Miao J, Xu L, Gao X, Zhang L, Li J, Gao H (2021). A stacking ensemble learning framework for genomic prediction. Front Genet.

[95] Ma Z, Wang J, Li J, Jia Y (2021). The association between obesity and problematic smartphone use among school-age children and adolescents: a cross-sectional study in Shanghai. BMC Public Health.

[96] Lee M, Kang D, Yoon J, Shim S, Kim IR, Oh D, Shin SY, Hesse BW, Cho J (2020). The difference in knowledge and attitudes of using mobile health applications between actual user and non-user among adults aged 50 and older. PLoS One.

[97] Dennison L, Morrison L, Conway G, Yardley L (2013). Opportunities and challenges for smartphone applications in supporting health behavior change: qualitative study. J Med Internet Res.

[98] Ernsting C, Stühmann LM, Dombrowski SU, Voigt-Antons JN, Kuhlmey A, Gellert P (2019). Associations of health app use and perceived effectiveness in people with cardiovascular diseases and diabetes: population-based survey. JMIR Mhealth Uhealth.

[99] Kaye L, Gondalia R, Thompson A, Stempel DA, Barrett MA (2021). The relationship between objective app engagement and medication adherence in asthma and COPD: a retrospective analysis. Sci Rep.

[100] Svendsen MT, Andersen F, Andersen KH, Pottegård A, Johannessen H, Möller S, August B, Feldman SR, Andersen KE (2018). A smartphone application supporting patients with psoriasis improves adherence to topical treatment: a randomized controlled trial. Br J Dermatol.

[101] Balato N, Megna M, Di Costanzo L, Balato A, Ayala F (2013). Educational and motivational support service: a pilot study for mobile-phone-based interventions in patients with psoriasis. Br J Dermatol.

[102] Zhu B, Wang Y, Zhou X, Cao C, Zong Y, Zhao X, Sha Z, Zhao X, Han S (2019). A controlled study of the feasibility and efficacy of a cloud-based interactive management program between patients with psoriasis and physicians. Med Sci Monit.

[103] McCloud T, Jones R, Lewis G, Bell V, Tsakanikos E (2020). Effectiveness of a mobile app intervention for anxiety and depression symptoms in university students: randomized controlled trial. JMIR Mhealth Uhealth.

[104] Rosenberg MA (2021). Trusting magic: interpretability of predictions from machine learning algorithms. Circulation.

[105] DMH_dataset. Synapse.

