# Supplementary figures and images for: Automated Machine Learning Analysis of Patients With Chronic Skin Disease Using a Medical Smartphone App: Retrospective Study
Source: J Med Internet Res. 2023 Nov 28;25:e50886. doi: 10.2196/50886 (PMC10716771; doi:10.2196/50886)

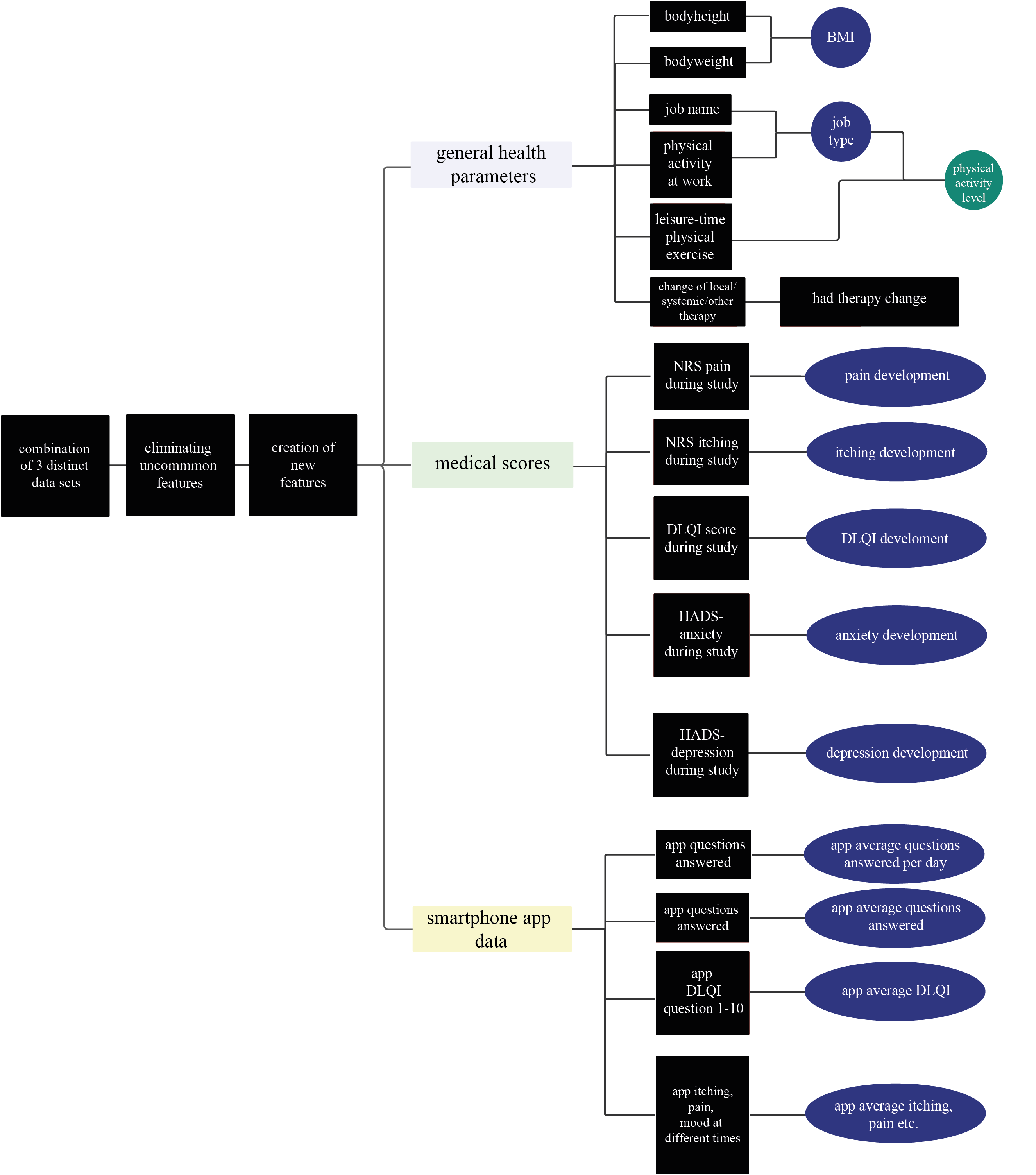

Supplement: Multimedia Appendix 1 [file jmir_v25i1e50886_app1.png]

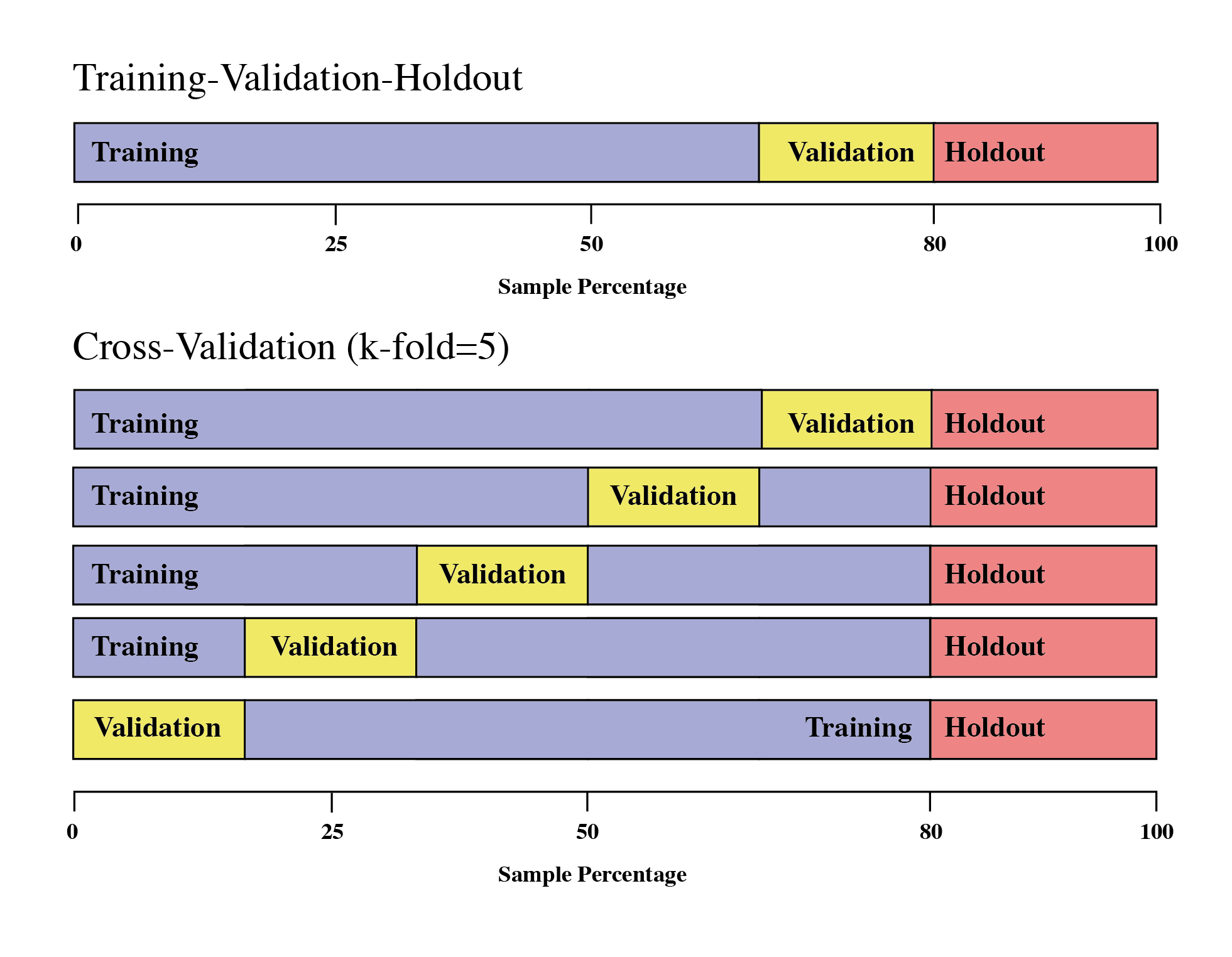

Supplement: Multimedia Appendix 5 [file jmir_v25i1e50886_app5.png]

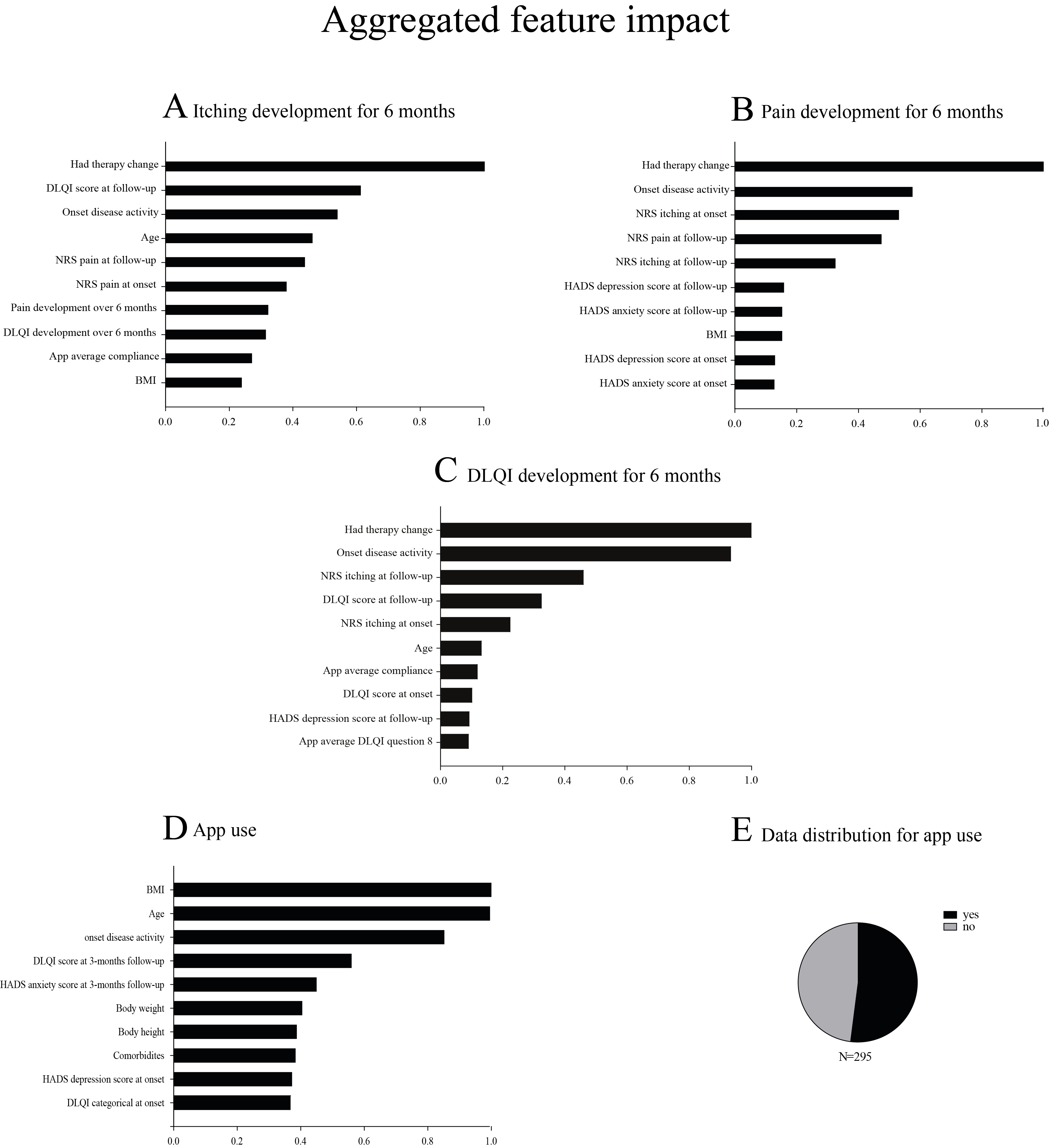

Supplement: Multimedia Appendix 8 [file jmir_v25i1e50886_app8.png]

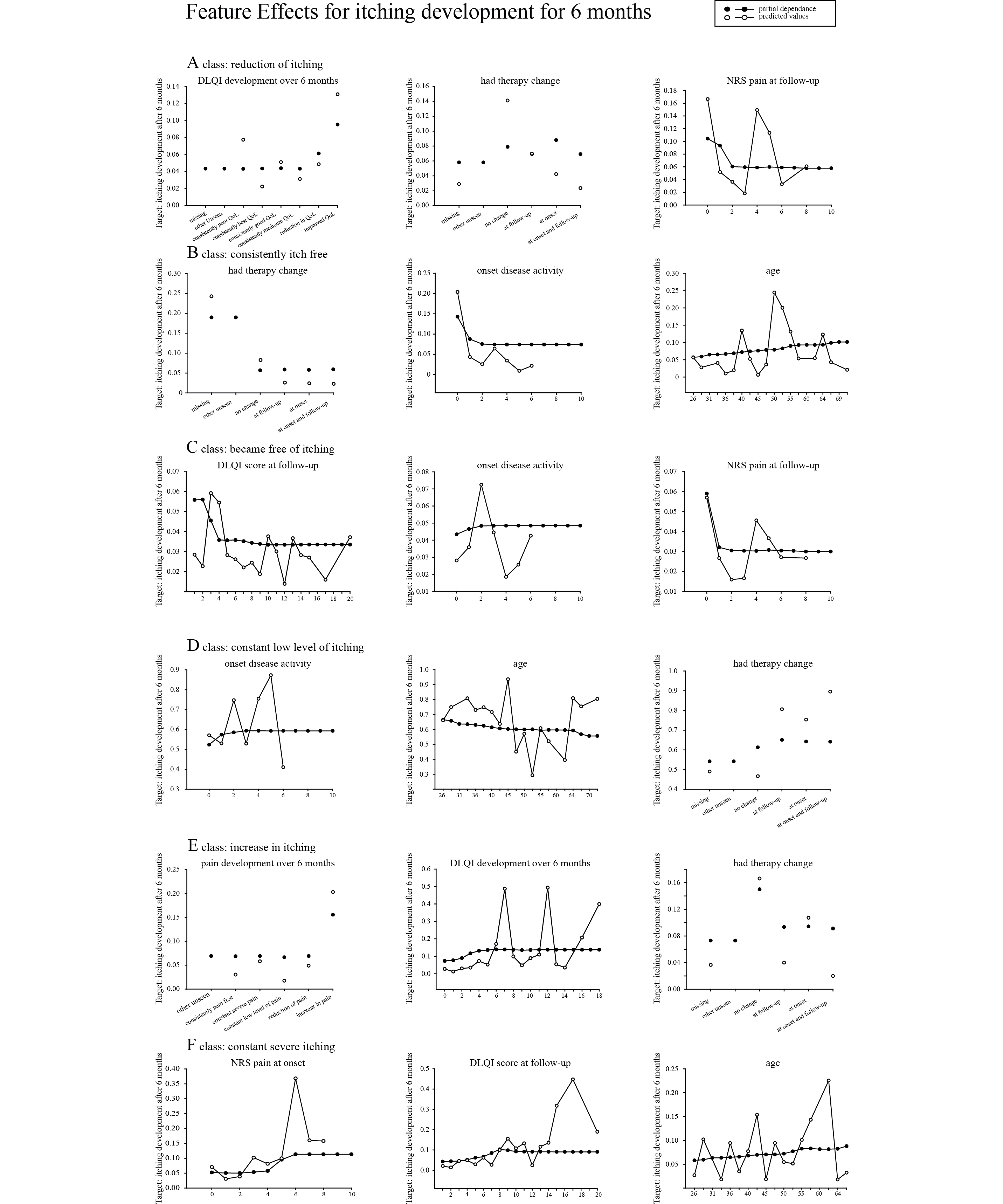

Supplement: Multimedia Appendix 9 [file jmir_v25i1e50886_app9.png]

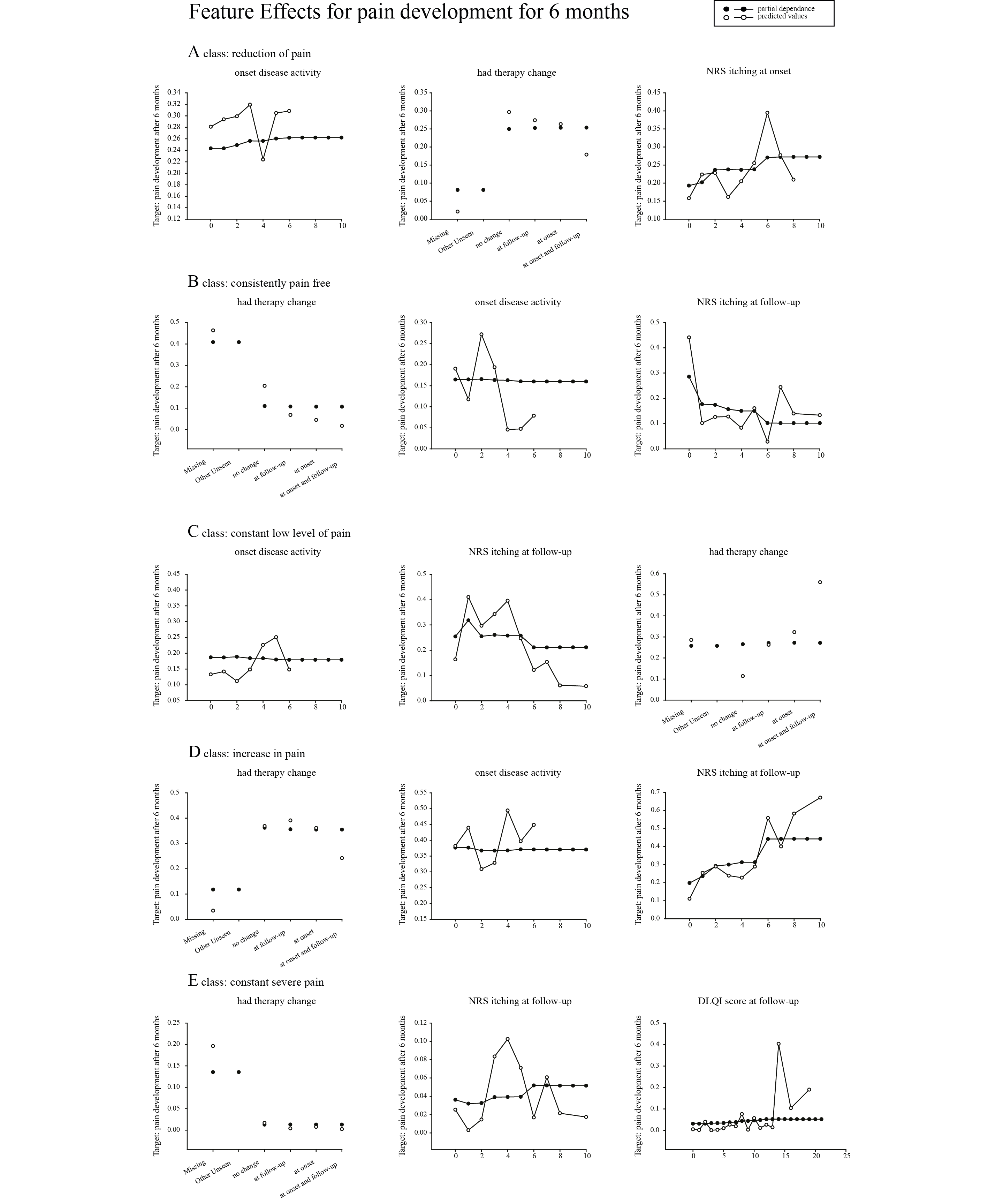

Supplement: Multimedia Appendix 10 [file jmir_v25i1e50886_app10.png]

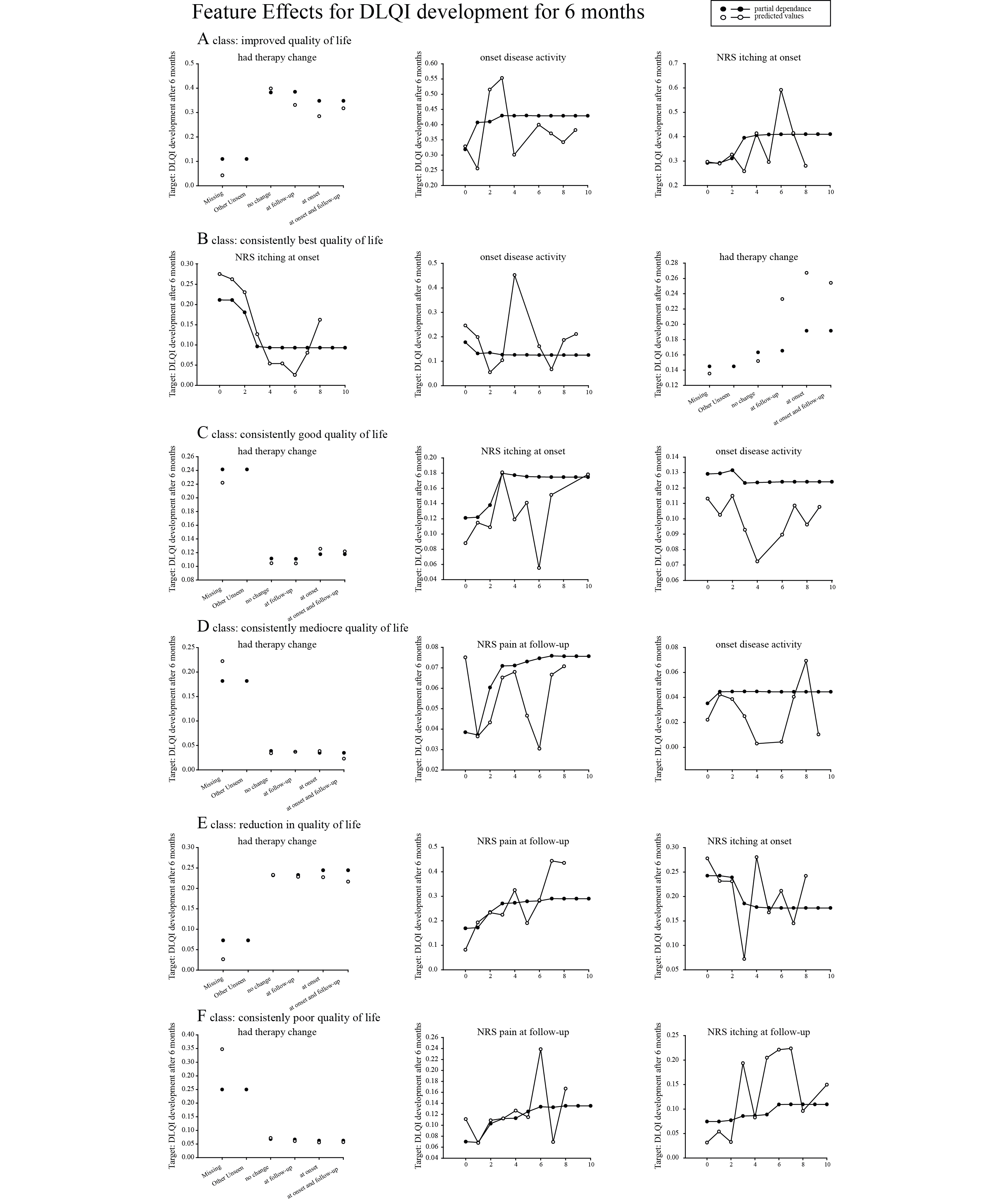

Supplement: Multimedia Appendix 11 [file jmir_v25i1e50886_app11.png]

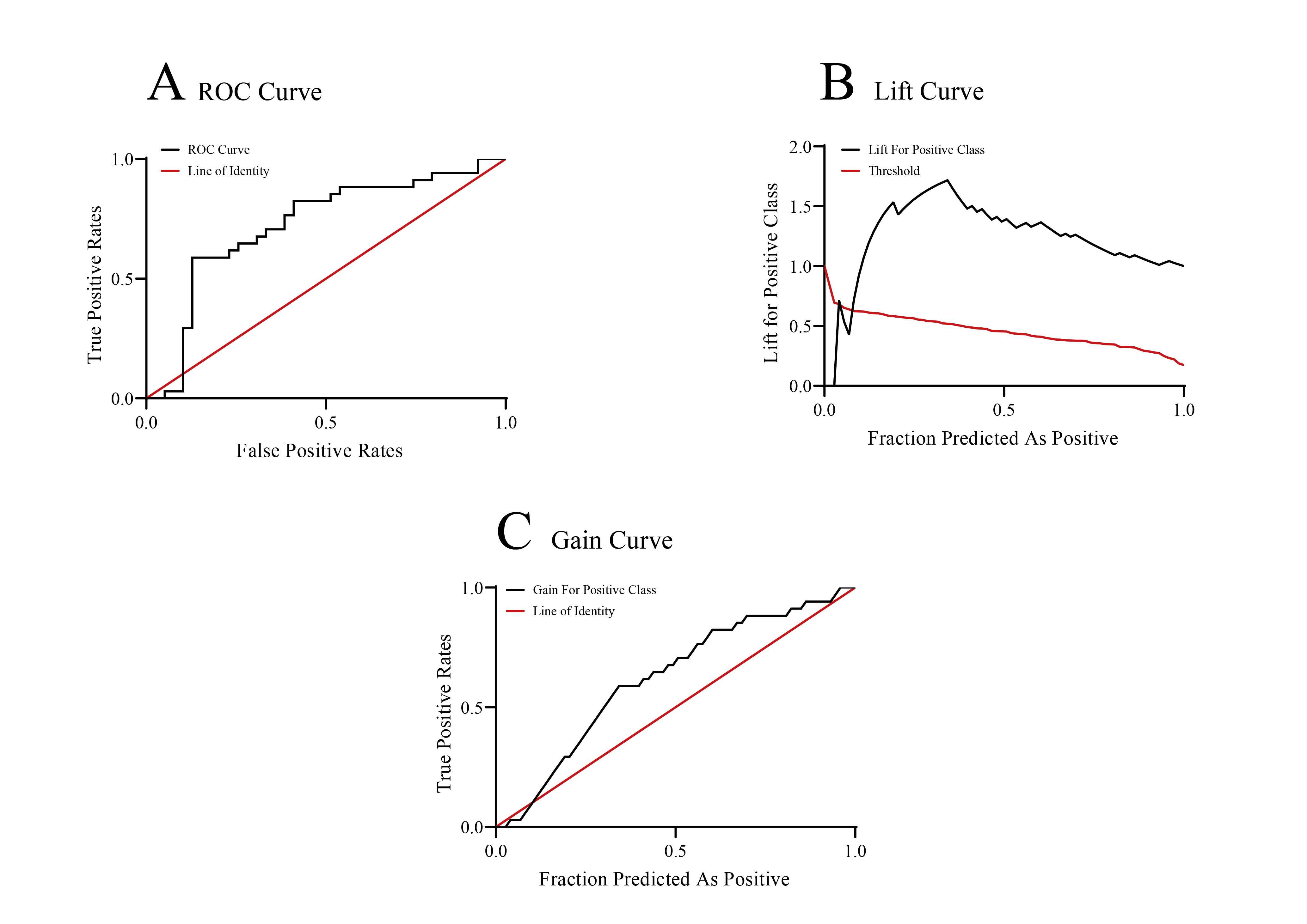

Supplement: Multimedia Appendix 12 [file jmir_v25i1e50886_app12.png]
