# Supplementary material for: Automated Machine Learning Analysis of Patients With Chronic Skin Disease Using a Medical Smartphone App: Retrospective Study
Source: J Med Internet Res. 2023 Nov 28;25:e50886. doi: 10.2196/50886 (PMC10716771; doi:10.2196/50886)
Supplement: Multimedia Appendix 2 [file jmir_v25i1e50886_app2.pdf]

new feature in secondary data set	feature type	method of classification
**body mass index (BMI)**	Numeric	body weight / body height * body height
**job type**	Multiclass	Manual categorization by the study investigators of a given patient's occupation into the following categories:<br><br>sedentary<br><br>physical occupation<br><br>student<br><br>retired<br><br>unemployed
**physical activity level at onset (0 months) and follow-up (3 & 6 months)**	Multiclass	Combination of type of occupation and leisure-time physical exercise with a cut-off time of 2 hours per week:<br><br>1. only exercise<br><br>2. only physical occupation<br><br>3. exercise and physical occupation<br><br>4. neither exercise nor physical occupation
**DLQI categorical at onset (0 months) and follow-up (3 & 6 months)**	Multiclass	Dermatological disease does not affect quality of life:<br>DLQI score = 0-1<br><br>Dermatological disease has only a minor impact on the quality of life:<br>DLQI score = 2-5<br><br>Dermatological disease has medium impact on quality of life:<br>DLQI score = 6-10<br><br>Dermatological disease has strong impact on quality of life:<br>DLQI score = 11-20<br><br>Dermatological disease has very strong impact on quality of life:<br>DLQI score = 21-30
**HADS anxiety categorical at onset (0 months) and follow-up (3 & 6 months)**	Multiclass	inconspicuous anxiety = 0-7<br><br>borderline anxiety = 8-10<br><br>highly suspected anxiety > 10
**HADS depression categorical at onset (0 months) and follow-up (3 & 6 months)**	Multiclass	inconspicuous depression = 0-7<br><br>borderline depression = 8-10<br><br>suspicious depression ≥ 11
**pain development for 6 months**	Multiclass	reduction of pain:<br>NRS pain at follow-up < NRS pain at 0 months<br><br>consistently pain free:<br>NRS pain = 0 at onset and follow-up<br><br>constant low level of pain:<br>NRS pain = 0-4 at onset and follow-up, if not 0 at both<br><br>increase in pain:<br>follow-up NRS pain > onset NRS pain

		constant severe pain:<br>NRS pain > 7 at onset and follow-up
**itching development for 6 months**	Multiclass	reduction of itching:<br>NRS itching at follow-up < NRS itching onset<br><br>consistently itch free:<br>NRS itching = 0 at onset and follow-up<br><br>became free of itching:<br>NRS itching = 0 at follow-up and > 1 at onset<br><br>constant low level of itching:<br>NRS itching 0-4 at onset and follow-up, if not 0 at both<br><br>increase in itching:<br>follow-up NRS itching > onset NRS itching<br><br>constant severe itching:<br>NRS itching > 7 at onset and follow-up
**DLQI development for 6 months**	Multiclass	improved quality of life:<br>onset DLQI score < follow-up DLQI score<br><br>consistently best quality of life:<br>DLQI score ≤ 1 at onset and follow-up<br><br>consistently good quality of life:<br>DLQI score > 1 and ≤ 5 at onset and follow-up<br><br>consistently mediocre quality of life<br>DLQI score > 5 and ≤ 10 at onset and follow-up<br><br>reduction in quality of life:<br>onset DLQI score > follow-up DLQI score<br><br>consistently poor quality of life:<br>DLQI score > 10 and ≤ 20 at onset and follow-up
**HADS anxiety development for 6 months**	Multiclass	increase in anxiety:<br>follow-up HADS-A > onset HADS-A<br><br>reduction in anxiety:<br>follow-up HADS-A < onset HADS-A<br><br>constant anxiety level:<br>onset HADS-A = 6 months HADS-A
**HADS depression development for 6 months**	Multiclass	increase in depression:<br>follow-up HADS-D > onset HADS-D<br><br>reduction in depression:<br>follow-up HADS-D < onset HADS-D<br><br>constant level of depression:<br>onset HADS-D = follow-up HADS-D
**had therapy change**	Multiclass	Categorization of features with local, systemic or other types of therapy:<br><br>no therapy change<br><br>therapy change at onset<br><br>therapy change at follow-up (after 6 months)

		therapy change at onset and follow-up (after 6 months)
**app usage (yes/no)**	Binary	Binary yes/no function depending on whether smartphone data export resulted in usage data per patient
**app days in use**	Numeric	number of days the smartphone app was used during the entire study period
**total answered app questions**	Numeric	number of questions answered in the smartphone app during the entire study period
**Categorized app questions answered per day**	Categorical	Categorization of average questions answered per day in the smartphone app throughout the study:<br><br>no questions answered:<br>average app questions answered per day = 0<br><br>low average number of app questions answered:<br>average app questions answered per day = 1-10<br><br>moderate average number of app questions answered:<br>average app questions answered per day = 11-30<br><br>high average number of app questions answered:<br>average app questions answered per day > 30
**app average questions answered per day**	Numeric	average number of questions answered per day in the smartphone app over the course of the study
**app average itching**	Numeric	average NRS itching score in the smartphone app throughout the study
**app average pain**	Numeric	average NRS pain score on the smartphone app throughout the study
**app average DLQI**	Numeric	average DLQI score on the smartphone app throughout the study
**app average compliance**	Numeric	average NRS patient compliance score on the smartphone app throughout the study
**app average tiredness/exhaustion**	Numeric	average NRS tiredness/exhaustion score on the smartphone app throughout the study
**app average mood**	Numeric	average NRS mood affection score in the smartphone app over the entire study period
**app average activities**	Numeric	average impaired activities score in the smartphone app over the entire study period
**app average morning stiffness**	Numeric	average NRS morning stiffness score on the smartphone app throughout the study
**app average morning stiffness duration**	Numeric	average duration of morning stiffness in the smartphone app over the study period
**average app sensitivity to touch or pressure (last 7d)**	Numeric	average NRS touch sensitivity score in the smartphone app throughout the study
**app average joint swelling (last 7d)**	Numeric	NRS average joint swelling score over 1 week documented on smartphone app

**app average joint pain (last 7d)**	Numeric	NRS average joint pain score over 1 week documented on smartphone app
**app average DLQI 1-10**	Numeric	average DLQI score for each DLQI 1-10 question in the smartphone app over the entire study period.
**app average DLQI total**	Numeric	average total DLQI score in the smartphone app over the entire study period
