# Supplementary material for: Automated Machine Learning Analysis of Patients With Chronic Skin Disease Using a Medical Smartphone App: Retrospective Study
Source: J Med Internet Res. 2023 Nov 28;25:e50886. doi: 10.2196/50886 (PMC10716771; doi:10.2196/50886)
Supplement: Multimedia Appendix 3 [file jmir_v25i1e50886_app3.docx]

# Target 1: Itching development for 6 months

## Features for Modeling and Summary Statistics

| **Feature Name** | **Var Type** | **Unique** | **Missing** | **Mean** | **Std Dev** | **Median** | **Min** | **Max** | **Target Leakage** |
| --- | --- | --- | --- | --- | --- | --- | --- | --- | --- |
| Gender | Categorical | 2 | 0 | N/A | N/A | N/A | N/A | N/A | Low |
| Age | Numeric | 54 | 0 | 49.89 | 13.34 | 52.0 | 23.0 | 84.0 | Low |
| Bodyheight | Numeric | 37 | 0 | 173.609 | 9.13 | 174.0 | 156.0 | 194.0 | Low |
| Bodyweight | Numeric | 70 | 0 | 86.9004 | 21.84 | 84.0 | 48.0 | 216.0 | Low |
| BMI | Numeric | 214 | 0 | 28.78 | 6.78 | 27.14 | 18.066 | 74.74 | Low |
| Nicotine | Numeric | 2 | 3 | 0.37 | 0.48 | 0.0 | 0.0 | 1.0 | Low |
| job type | Categorical | 5 | 2 | N/A | N/A | N/A | N/A | N/A | Low |
| Comorbidities | Categorical | 97 | 38 | N/A | N/A | N/A | N/A | N/A | Low |
| Leisure-time physical exercise with a cut-off time of 2 hours per week (1=yes; 0=no) | Numeric | 2 | 3 | 0.48 | 0.5 | 0.0 | 0.0 | 1.0 | Low |
| Physical activity level at onset | Categorical | 4 | 0 | N/A | N/A | N/A | N/A | N/A | Low |
| NRS pain at onset | Numeric | 11 | 0 | 1.88 | 2.24 | 1.0 | 0.0 | 10.0 | Low |
| Onset disease activity | Numeric | 11 | 108 | 2.7008 | 2.53 | 2.0 | 0.0 | 10.0 | Low |
| DLQI score at onset | Numeric | 30 | 0 | 6.97 | 7.11 | 4.0 | 0.0 | 30.0 | Low |
| HADS anxiety score at onset | Numeric | 19 | 0 | 6.23 | 4.28 | 5.0 | 0.0 | 20.0 | Low |
| HADS depression score at onset | Numeric | 19 | 0 | 4.52 | 4.13 | 3.0 | 0.0 | 19.0 | Low |
| DLQI categorial at onset | Categorical | 4 | 0 | N/A | N/A | N/A | N/A | N/A | Low |
| HADS anxiety categorial at onset | Categorical | 3 | 0 | N/A | N/A | N/A | N/A | N/A | Low |
| HADS depression categorial at onset | Categorical | 3 | 0 | N/A | N/A | N/A | N/A | N/A | Low |
| NRS pain at follow-up | Numeric | 11 | 0 | 2.37 | 2.602 | 1.0 | 0.0 | 10.0 | Low |
| DLQI score at follow-up | Numeric | 25 | 1 | 5.4 | 5.93 | 3.0 | 0.0 | 33.0 | Low |
| HADS anxiety score at follow-up | Numeric | 18 | 0 | 5.65 | 4.21 | 5.0 | 0.0 | 20.0 | Low |
| HADS depression score at follow-up | Numeric | 18 | 0 | 4.19 | 4.14 | 3.0 | 0.0 | 19.0 | Low |
| DLQI categorical at follow-up | Categorical | 4 | 1 | N/A | N/A | N/A | N/A | N/A | Low |
| HADS anxiety categorical at follow-up | Categorical | 3 | 1 | N/A | N/A | N/A | N/A | N/A | Low |
| HADS depression categorical at follow-up | Categorical | 3 | 1 | N/A | N/A | N/A | N/A | N/A | Low |
| pain development over 6 months | Categorical | 5 | 0 | N/A | N/A | N/A | N/A | N/A | Low |
| DLQI development over 6 months | Categorical | 6 | 1 | N/A | N/A | N/A | N/A | N/A | Low |
| HADS anxiety development over 6 months | Categorical | 5 | 1 | N/A | N/A | N/A | N/A | N/A | Low |
| HADS depression development over 6 months | Categorical | 5 | 1 | N/A | N/A | N/A | N/A | N/A | Low |
| had therapy change | Categorical | 4 | 49 | N/A | N/A | N/A | N/A | N/A | Low |
| app usage | Categorical | 2 | 0 | N/A | N/A | N/A | N/A | N/A | Low |
| app days in use | Numeric | 52 | 0 | 12.502 | 25.28 | 4.0 | 0.0 | 218.0 | Low |
| Categorized app questions answered per day | Categorical | 4 | 0 | N/A | N/A | N/A | N/A | N/A | Low |
| app answered questions per day | Numeric | 104 | 0 | 10.85 | 21.29 | 0.0 | 0.0 | 217.32 | Low |
| total answered app questions | Numeric | 86 | 0 | 328.85 | 1048.013 | 0.0 | 0.0 | 10268.0 | Low |
| app average pain | Numeric | 81 | 122 | 2.19 | 2.32 | 1.33 | 0.0 | 8.96 | Low |
| app average Itching | Numeric | 74 | 137 | 2.19 | 2.19 | 1.59 | 0.0 | 9.96 | Low |
| app average DLQI | Numeric | 106 | 122 | 34.74 | 24.58 | 33.0 | 0.0 | 99.29 | Low |
| app average compliance | Numeric | 41 | 171 | 7.091 | 3.23 | 8.403 | 0.0 | 10.0 | Low |
| app average tiredness/exhaustion | Numeric | 37 | 180 | 3.68 | 2.54 | 3.47 | 0.0 | 9.17 | Low |
| app average mood | Numeric | 82 | 122 | 2.85 | 2.63 | 2.082 | 0.0 | 9.905 | Low |
| app average activities | Numeric | 81 | 122 | 2.66 | 2.509 | 2.0 | 0.0 | 9.88 | Low |
| app average morning stiffness | Numeric | 39 | 177 | 3.55 | 3.095 | 3.69 | 0.0 | 9.0 | Low |
| app average morning stiffness duration | Numeric | 65 | 139 | 2.92 | 2.035 | 2.705 | 0.0 | 9.0 | Low |
| app average sensitivity to touch or pressure (last 7d) | Numeric | 36 | 179 | 3.36 | 2.89 | 3.28 | 0.0 | 9.5 | Low |
| app average joint swelling (last 7d) | Numeric | 38 | 178 | 2.7 | 2.504 | 2.13 | 0.0 | 9.0 | Low |
| app average joint pain (last 7d) | Numeric | 38 | 179 | 3.82 | 2.501 | 4.0 | 0.0 | 8.55 | Low |
| app average DLQI total | Numeric | 46 | 187 | 6.75 | 5.82 | 5.409 | 0.0 | 21.38 | Low |

## Data Quality Handling Report

| **Feature Name** | **Var Type** | **Missing Count** | **Missing Percentage** | **Imputation Name** | **Imputation Description** |
| --- | --- | --- | --- | --- | --- |
| app average DLQI total | Numeric | 234 | 80 | Missing Values Imputed | Missing indicator treated as feature, Imputed value: 5.8488 |
| app average tiredness/exhaustion | Numeric | 217 | 74 | Missing Values Imputed | Missing indicator treated as feature, Imputed value: 3.5 |
| app average sensitivity to touch or pressure (last 7d) | Numeric | 217 | 74 | Missing Values Imputed | Missing indicator treated as feature, Imputed value: 3.1895 |
| app average joint pain (last 7d) | Numeric | 217 | 74 | Missing Values Imputed | Missing indicator treated as feature, Imputed value: 4 |
| app average joint swelling (last 7d) | Numeric | 216 | 74 | Missing Values Imputed | Missing indicator treated as feature, Imputed value: 2.1333 |
| app average morning stiffness | Numeric | 215 | 73 | Missing Values Imputed | Missing indicator treated as feature, Imputed value: 3.4615 |
| app average compliance | Numeric | 208 | 71 | Missing Values Imputed | Missing indicator treated as feature, Imputed value: 8.3333 |
| app average morning stiffness duration | Numeric | 167 | 57 | Missing Values Imputed | Missing indicator treated as feature, Imputed value: 2.75 |
| app average Itching | Numeric | 166 | 57 | Missing Values Imputed | Missing indicator treated as feature, Imputed value: 1.8333 |
| app average pain | Numeric | 147 | 50 | Missing Values Imputed | Missing indicator treated as feature, Imputed value: 1.3333 |
| app average DLQI | Numeric | 147 | 50 | Missing Values Imputed | Imputed value: 33.6984 |
| app average mood | Numeric | 147 | 50 | Missing Values Imputed | Imputed value: 2.4286 |
| app average activities | Numeric | 147 | 50 | Missing Values Imputed | Imputed value: 2 |
| Onset disease activity | Numeric | 138 | 47 | Missing Values Imputed | Missing indicator treated as feature, Imputed value: 2 |
| had therapy change | Categorical | 59 | 20 | Ordinal encoding of categorical variables | Imputed value: -2 |
| Comorbidities | Categorical | 46 | 16 | Ordinal encoding of categorical variables | Imputed value: -2 |
| Nicotine | Numeric | 4 | 1 | Missing Values Imputed | Missing indicator treated as feature, Imputed value: 0 |
| Leisure-time physical exercise with a cut-off time of 2 hours per week (1=yes; 0=no) | Numeric | 4 | 1 | Missing Values Imputed | Missing indicator treated as feature, Imputed value: 0 |
| job type | Categorical | 3 | 1 | Ordinal encoding of categorical variables | Imputed value: -2 |
| DLQI categorical at follow-up | Categorical | 2 | 1 | Ordinal encoding of categorical variables | Imputed value: -2 |
| HADS anxiety categorical at follow-up | Categorical | 2 | 1 | Ordinal encoding of categorical variables | Imputed value: -2 |
| HADS depression categorical at follow-up | Categorical | 2 | 1 | Ordinal encoding of categorical variables | Imputed value: -2 |
| DLQI development over 6 months | Categorical | 2 | 1 | Ordinal encoding of categorical variables | Imputed value: -2 |
| HADS anxiety development over 6 months | Categorical | 2 | 1 | Ordinal encoding of categorical variables | Imputed value: -2 |
| HADS depression development over 6 months | Categorical | 2 | 1 | Ordinal encoding of categorical variables | Imputed value: -2 |
| DLQI score at follow-up | Numeric | 2 | 1 | Missing Values Imputed | Missing indicator treated as feature, Imputed value: 3 |
| Bodyheight | Numeric | 1 | 0 | Missing Values Imputed | Missing indicator treated as feature, Imputed value: 174 |
| Bodyweight | Numeric | 1 | 0 | Missing Values Imputed | Imputed value: 85 |
| BMI | Numeric | 1 | 0 | Missing Values Imputed | Imputed value: 27.4286 |
| HADS anxiety score at follow-up | Numeric | 1 | 0 | Missing Values Imputed | Missing indicator treated as feature, Imputed value: 5 |
| HADS depression score at follow-up | Numeric | 1 | 0 | Missing Values Imputed | Imputed value: 3 |
| Gender | Categorical | 0 | 0 | Ordinal encoding of categorical variables | Imputed value: -2 |
| Physical activity level at onset | Categorical | 0 | 0 | Ordinal encoding of categorical variables | Imputed value: -2 |
| DLQI categorial at onset | Categorical | 0 | 0 | Ordinal encoding of categorical variables | Imputed value: -2 |
| HADS anxiety categorial at onset | Categorical | 0 | 0 | Ordinal encoding of categorical variables | Imputed value: -2 |
| HADS depression categorial at onset | Categorical | 0 | 0 | Ordinal encoding of categorical variables | Imputed value: -2 |
| pain development over 6 months | Categorical | 0 | 0 | Ordinal encoding of categorical variables | Imputed value: -2 |
| app usage | Categorical | 0 | 0 | Ordinal encoding of categorical variables | Imputed value: -2 |
| Categorized app questions answered per day | Categorical | 0 | 0 | Ordinal encoding of categorical variables | Imputed value: -2 |
| Age | Numeric | 0 | 0 | Missing Values Imputed | Imputed value: 52 |
| NRS pain at onset | Numeric | 0 | 0 | Missing Values Imputed | Imputed value: 1 |
| DLQI score at onset | Numeric | 0 | 0 | Missing Values Imputed | Imputed value: 4 |
| HADS anxiety score at onset | Numeric | 0 | 0 | Missing Values Imputed | Imputed value: 5 |
| HADS depression score at onset | Numeric | 0 | 0 | Missing Values Imputed | Imputed value: 3 |
| NRS pain at follow-up | Numeric | 0 | 0 | Missing Values Imputed | Imputed value: 1 |
| app days in use | Numeric | 0 | 0 | Missing Values Imputed | Imputed value: 4 |
| app answered questions per day | Numeric | 0 | 0 | Missing Values Imputed | Imputed value: 0 |
| total answered app questions | Numeric | 0 | 0 | Missing Values Imputed | Imputed value: 0 |

## Cross Validation Scores

| **Fold** | **Cross Validation Score (LogLoss)** |
| --- | --- |
| Fold 1 | 0.93017 |
| Fold 2 | 1.0925 |
| Fold 3 | 0.96708 |
| Fold 4 | 1.09005 |
| Fold 5 | 1.01694 |

# Target 2: Pain development for 6 months

## Features for Modeling and Summary Statistics

| **Feature Name** | **Var Type** | **Unique** | **Missing** | **Mean** | **Std Dev** | **Median** | **Min** | **Max** | **Target Leakage** |
| --- | --- | --- | --- | --- | --- | --- | --- | --- | --- |
| Age | Numeric | 54 | 0 | 49.89 | 13.34 | 52.0 | 23.0 | 84.0 | Low |
| Bodyheight | Numeric | 37 | 0 | 173.609 | 9.13 | 174.0 | 156.0 | 194.0 | Low |
| Bodyweight | Numeric | 70 | 0 | 86.9004 | 21.84 | 84.0 | 48.0 | 216.0 | Low |
| BMI | Numeric | 214 | 0 | 28.78 | 6.78 | 27.14 | 18.066 | 74.74 | Low |
| Nicotine | Numeric | 2 | 3 | 0.37 | 0.48 | 0.0 | 0.0 | 1.0 | Low |
| NRS itching at onset | Numeric | 11 | 0 | 2.6 | 2.46 | 2.0 | 0.0 | 10.0 | Low |
| Onset disease activity | Numeric | 11 | 108 | 2.7008 | 2.53 | 2.0 | 0.0 | 10.0 | Low |
| DLQI score at onset | Numeric | 30 | 0 | 6.97 | 7.11 | 4.0 | 0.0 | 30.0 | Low |
| DLQI categorial at onset | Categorical | 4 | 0 | N/A | N/A | N/A | N/A | N/A | Low |
| NRS itching at follow-up | Numeric | 11 | 0 | 2.62 | 2.602 | 2.0 | 0.0 | 10.0 | Low |
| DLQI score at follow-up | Numeric | 25 | 1 | 5.4 | 5.93 | 3.0 | 0.0 | 33.0 | Low |
| HADS depression score at follow-up | Numeric | 18 | 0 | 4.19 | 4.14 | 3.0 | 0.0 | 19.0 | Low |
| DLQI categorical at follow-up | Categorical | 4 | 1 | N/A | N/A | N/A | N/A | N/A | Low |
| pain development over 6 months | Categorical | 5 | 0 | N/A | N/A | N/A | N/A | N/A | N/A |
| had therapy change | Categorical | 4 | 49 | N/A | N/A | N/A | N/A | N/A | Low |
| app days in use | Numeric | 52 | 0 | 12.502 | 25.28 | 4.0 | 0.0 | 218.0 | Low |
| app answered questions per day | Numeric | 104 | 0 | 10.85 | 21.29 | 0.0 | 0.0 | 217.32 | Low |
| total answered app questions | Numeric | 86 | 0 | 328.85 | 1048.013 | 0.0 | 0.0 | 10268.0 | Low |
| app average compliance | Numeric | 41 | 171 | 7.091 | 3.23 | 8.403 | 0.0 | 10.0 | Low |
| app average morning stiffness duration | Numeric | 65 | 139 | 2.92 | 2.035 | 2.705 | 0.0 | 9.0 | Low |
| app average DLQI 6 | Numeric | 31 | 186 | 0.75 | 0.86 | 0.57 | 0.0 | 3.0 | Low |
| app average DLQI 7 | Numeric | 13 | 195 | 0.205 | 0.52 | 0.0 | 0.0 | 3.0 | Low |
| app average DLQI 7a | Numeric | 18 | 194 | 0.37 | 0.54 | 0.0 | 0.0 | 1.88 | Low |
| app average DLQI 8 | Numeric | 25 | 189 | 0.6 | 0.73 | 0.26 | 0.0 | 2.75 | Low |

## Data Quality Handling Report

| **Feature Name** | **Var Type** | **Missing Count** | **Missing Percentage** | **Imputation Name** | **Imputation Description** |
| --- | --- | --- | --- | --- | --- |
| app average DLQI 7 | Numeric | 242 | 83 | Missing Values Imputed | Missing indicator treated as feature, Imputed value: 0 |
| app average DLQI 7a | Numeric | 241 | 82 | Missing Values Imputed | Missing indicator treated as feature, Imputed value: 0 |
| app average DLQI 8 | Numeric | 236 | 81 | Missing Values Imputed | Missing indicator treated as feature, Imputed value: 0.3333 |
| app average DLQI 6 | Numeric | 234 | 80 | Missing Values Imputed | Missing indicator treated as feature, Imputed value: 0.6 |
| app average compliance | Numeric | 208 | 71 | Missing Values Imputed | Missing indicator treated as feature, Imputed value: 8.3333 |
| app average morning stiffness duration | Numeric | 167 | 57 | Missing Values Imputed | Missing indicator treated as feature, Imputed value: 2.75 |
| Onset disease activity | Numeric | 138 | 47 | Missing Values Imputed | Missing indicator treated as feature, Imputed value: 2 |
| had therapy change | Categorical | 59 | 20 | Ordinal encoding of categorical variables | Imputed value: -2 |
| Nicotine | Numeric | 4 | 1 | Missing Values Imputed | Missing indicator treated as feature, Imputed value: 0 |
| DLQI categorical at follow-up | Categorical | 2 | 1 | Ordinal encoding of categorical variables | Imputed value: -2 |
| DLQI score at follow-up | Numeric | 2 | 1 | Missing Values Imputed | Missing indicator treated as feature, Imputed value: 3 |
| Bodyheight | Numeric | 1 | 0 | Missing Values Imputed | Missing indicator treated as feature, Imputed value: 174 |
| Bodyweight | Numeric | 1 | 0 | Missing Values Imputed | Imputed value: 85 |
| BMI | Numeric | 1 | 0 | Missing Values Imputed | Imputed value: 27.4286 |
| HADS depression score at follow-up | Numeric | 1 | 0 | Missing Values Imputed | Missing indicator treated as feature, Imputed value: 3 |
| DLQI categorial at onset | Categorical | 0 | 0 | Ordinal encoding of categorical variables | Imputed value: -2 |
| Age | Numeric | 0 | 0 | Missing Values Imputed | Imputed value: 52 |
| NRS itching at onset | Numeric | 0 | 0 | Missing Values Imputed | Imputed value: 2 |
| DLQI score at onset | Numeric | 0 | 0 | Missing Values Imputed | Imputed value: 4 |
| NRS itching at follow-up | Numeric | 0 | 0 | Missing Values Imputed | Imputed value: 2 |
| app days in use | Numeric | 0 | 0 | Missing Values Imputed | Imputed value: 4 |
| app answered questions per day | Numeric | 0 | 0 | Missing Values Imputed | Imputed value: 0 |
| total answered app questions | Numeric | 0 | 0 | Missing Values Imputed | Imputed value: 0 |

## Cross Validation Scores

| **Fold** | **Cross Validation Score (LogLoss)** |
| --- | --- |
| Fold 1 | 1.17986 |
| Fold 2 | 1.15482 |
| Fold 3 | 1.13372 |
| Fold 4 | 1.16874 |
| Fold 5 | 1.14314 |

# Target 3: DLQI development for 6 months

## Features for Modeling and Summary Statistics

| **Feature Name** | **Var Type** | **Unique** | **Missing** | **Mean** | **Std Dev** | **Median** | **Min** | **Max** | **Target Leakage** |
| --- | --- | --- | --- | --- | --- | --- | --- | --- | --- |
| Age | Numeric | 56 | 0 | 49.61 | 13.6 | 52.0 | 20.0 | 84.0 | Low |
| Bodyheight | Numeric | 38 | 1 | 173.79 | 8.96 | 175.0 | 154.0 | 193.0 | Low |
| Bodyweight | Numeric | 71 | 1 | 86.55 | 20.53 | 84.0 | 48.0 | 180.0 | Low |
| BMI | Numeric | 219 | 1 | 28.59 | 6.13 | 27.16 | 18.066 | 54.4 | Low |
| job type | Categorical | 5 | 3 | N/A | N/A | N/A | N/A | N/A | Low |
| NRS pain at onset | Numeric | 11 | 2 | 1.82 | 2.18 | 1.0 | 0.0 | 10.0 | Low |
| NRS itching at onset | Numeric | 11 | 2 | 2.64 | 2.48 | 2.0 | 0.0 | 10.0 | Low |
| Onset disease activity | Numeric | 11 | 110 | 2.64 | 2.58 | 2.0 | 0.0 | 10.0 | Low |
| HADS anxiety score at onset | Numeric | 19 | 0 | 6.18 | 4.23 | 5.5 | 0.0 | 20.0 | Low |
| HADS depression score at onset | Numeric | 19 | 0 | 4.43 | 4.033 | 3.0 | 0.0 | 19.0 | Low |
| NRS pain at follow-up | Numeric | 11 | 5 | 2.36 | 2.64 | 1.0 | 0.0 | 10.0 | Low |
| NRS itching at follow-up | Numeric | 11 | 5 | 2.63 | 2.59 | 2.0 | 0.0 | 10.0 | Low |
| HADS anxiety score at follow-up | Numeric | 19 | 0 | 5.704 | 4.31 | 5.0 | 0.0 | 20.0 | Low |
| HADS depression score at follow-up | Numeric | 18 | 0 | 4.3 | 4.207 | 3.0 | 0.0 | 19.0 | Low |
| pain development over 6 months | Categorical | 5 | 7 | N/A | N/A | N/A | N/A | N/A | Low |
| 6_months_itching_change | Categorical | 7 | 7 | N/A | N/A | N/A | N/A | N/A | Low |
| DLQI development over 6 months | Categorical | 6 | 0 | N/A | N/A | N/A | N/A | N/A | N/A |
| HADS depression development over 6 months | Categorical | 5 | 0 | N/A | N/A | N/A | N/A | N/A | Low |
| had therapy change | Categorical | 4 | 45 | N/A | N/A | N/A | N/A | N/A | Low |
| app days in use | Numeric | 50 | 0 | 12.25 | 24.302 | 4.0 | 0.0 | 218.0 | Low |
| app answered questions per day | Numeric | 109 | 0 | 10.28 | 16.8 | 0.0 | 0.0 | 97.86 | Low |
| app average mood | Numeric | 84 | 121 | 2.95 | 2.68 | 2.43 | 0.0 | 10.0 | Low |
| app average sensitivity to touch or pressure (last 7d) | Numeric | 37 | 178 | 3.32 | 2.98 | 3.0 | 0.0 | 10.0 | Low |

## Data Quality Handling Report

| **Feature Name** | **Var Type** | **Missing Count** | **Missing Percentage** | **Imputation Name** | **Imputation Description** |
| --- | --- | --- | --- | --- | --- |
| app average sensitivity to touch or pressure (last 7d) | Numeric | 220 | 74 | Missing Values Imputed | Missing indicator treated as feature, Imputed value: 3 |
| app average mood | Numeric | 148 | 50 | Missing Values Imputed | Missing indicator treated as feature, Imputed value: 2.4286 |
| Onset disease activity | Numeric | 141 | 47 | Missing Values Imputed | Missing indicator treated as feature, Imputed value: 2 |
| had therapy change | Categorical | 59 | 20 | Ordinal encoding of categorical variables | Imputed value: -2 |
| pain development over 6 months | Categorical | 8 | 3 | Ordinal encoding of categorical variables | Imputed value: -2 |
| 6_months_itching_change | Categorical | 8 | 3 | Ordinal encoding of categorical variables | Imputed value: -2 |
| NRS pain at follow-up | Numeric | 6 | 2 | Missing Values Imputed | Missing indicator treated as feature, Imputed value: 1 |
| NRS itching at follow-up | Numeric | 6 | 2 | Missing Values Imputed | Imputed value: 2 |
| job type | Categorical | 3 | 1 | Ordinal encoding of categorical variables | Imputed value: -2 |
| NRS pain at onset | Numeric | 2 | 1 | Missing Values Imputed | Missing indicator treated as feature, Imputed value: 1 |
| NRS itching at onset | Numeric | 2 | 1 | Missing Values Imputed | Imputed value: 2 |
| Bodyheight | Numeric | 1 | 0 | Missing Values Imputed | Missing indicator treated as feature, Imputed value: 174 |
| Bodyweight | Numeric | 1 | 0 | Missing Values Imputed | Imputed value: 84 |
| BMI | Numeric | 1 | 0 | Missing Values Imputed | Imputed value: 27.4653 |
| HADS depression development over 6 months | Categorical | 0 | 0 | Ordinal encoding of categorical variables | Imputed value: -2 |
| Age | Numeric | 0 | 0 | Missing Values Imputed | Imputed value: 52 |
| HADS anxiety score at onset | Numeric | 0 | 0 | Missing Values Imputed | Imputed value: 5 |
| HADS depression score at onset | Numeric | 0 | 0 | Missing Values Imputed | Imputed value: 3 |
| HADS anxiety score at follow-up | Numeric | 0 | 0 | Missing Values Imputed | Imputed value: 5 |
| HADS depression score at follow-up | Numeric | 0 | 0 | Missing Values Imputed | Imputed value: 3 |
| app days in use | Numeric | 0 | 0 | Missing Values Imputed | Imputed value: 4 |
| app answered questions per day | Numeric | 0 | 0 | Missing Values Imputed | Imputed value: 0.625 |

## Cross Validation Scores

| **Fold** | **Cross Validation Score (LogLoss)** |
| --- | --- |
| Fold 1 | 1.40649 |
| Fold 2 | 1.2832 |
| Fold 3 | 1.42287 |
| Fold 4 | 1.19464 |
| Fold 5 | 1.51796 |

# Target 4: app usage

## Features for Modeling and Summary Statistics

| **Feature Name** | **Var Type** | **Unique** | **Missing** | **Mean** | **Std Dev** | **Median** | **Min** | **Max** | **Target Leakage** |
| --- | --- | --- | --- | --- | --- | --- | --- | --- | --- |
| Gender | Categorical | 2 | 0 | N/A | N/A | N/A | N/A | N/A | Low |
| Age | Numeric | 58 | 0 | 50.69 | 13.102 | 53.0 | 21.0 | 84.0 | Low |
| Bodyheight | Numeric | 41 | 2 | 173.49 | 9.13 | 174.0 | 152.0 | 194.0 | Low |
| Bodyweight | Numeric | 80 | 2 | 88.13 | 23.0098 | 85.0 | 15.0 | 216.0 | Low |
| BMI | Numeric | 271 | 2 | 29.25 | 6.85 | 28.0 | 18.066 | 74.74 | Low |
| Nicotine | Numeric | 2 | 5 | 0.36 | 0.48 | 0.0 | 0.0 | 1.0 | Low |
| job type | Categorical | 5 | 6 | N/A | N/A | N/A | N/A | N/A | Low |
| Comorbidities | Text | 134 | 45 | N/A | N/A | N/A | N/A | N/A | N/A |
| leisure time physical exercise with a cut off time of 2 hours per week (1=yes; 0=no) | Numeric | 2 | 6 | 0.46 | 0.5 | 0.0 | 0.0 | 1.0 | Low |
| physical activity level at onset | Categorical | 4 | 0 | N/A | N/A | N/A | N/A | N/A | Low |
| NRS pain at onset | Numeric | 11 | 4 | 2.19 | 2.39 | 1.0 | 0.0 | 10.0 | Low |
| NRS itching at onset | Numeric | 11 | 4 | 2.73 | 2.59 | 2.0 | 0.0 | 10.0 | Low |
| onset disease activity | Numeric | 11 | 131 | 2.93 | 2.68 | 2.0 | 0.0 | 10.0 | Low |
| DLQI score at onset | Numeric | 30 | 2 | 7.35 | 7.702 | 5.0 | 0.0 | 30.0 | Low |
| HADS anxiety score at onset | Numeric | 20 | 2 | 6.28 | 4.33 | 6.0 | 0.0 | 20.0 | Low |
| HADS depression score at onset | Numeric | 18 | 2 | 4.68 | 4.28 | 4.0 | 0.0 | 19.0 | Low |
| DLQI categorial at onset | Categorical | 4 | 2 | N/A | N/A | N/A | N/A | N/A | Low |
| HADS anxiety categorial at onset | Categorical | 3 | 2 | N/A | N/A | N/A | N/A | N/A | Low |
| HADS depression categorial at onset | Categorical | 3 | 2 | N/A | N/A | N/A | N/A | N/A | Low |
| NRS pain at follow-up | Numeric | 11 | 65 | 2.34 | 2.54 | 1.0 | 0.0 | 10.0 | Low |
| NRS itching at follow-up | Numeric | 11 | 65 | 2.55 | 2.55 | 1.0 | 0.0 | 10.0 | Low |
| DLQI score at follow-up | Numeric | 26 | 61 | 5.39 | 6.23 | 3.0 | 0.0 | 33.0 | Low |
| HADS anxiety score at follow-up | Numeric | 19 | 60 | 5.51 | 4.048 | 5.0 | 0.0 | 20.0 | Low |
| HADS depression score at follow-up | Numeric | 17 | 60 | 4.209 | 4.056 | 3.0 | 0.0 | 18.0 | Low |
| DLQI categorical at follow-up | Categorical | 4 | 61 | N/A | N/A | N/A | N/A | N/A | Low |
| HADS anxiety categorical at follow-up | Categorical | 3 | 61 | N/A | N/A | N/A | N/A | N/A | Low |
| HADS depression categorical at follow-up | Categorical | 3 | 61 | N/A | N/A | N/A | N/A | N/A | Low |
| pain development over 6 months | Categorical | 5 | 67 | N/A | N/A | N/A | N/A | N/A | Low |
| itching development over 6 months | Categorical | 6 | 67 | N/A | N/A | N/A | N/A | N/A | Low |
| DLQI development over 6 months | Categorical | 6 | 61 | N/A | N/A | N/A | N/A | N/A | Low |
| HADS anxiety development over 6 months | Categorical | 5 | 61 | N/A | N/A | N/A | N/A | N/A | Low |
| HADS depression development over 6 months | Categorical | 5 | 61 | N/A | N/A | N/A | N/A | N/A | Low |
| had therapy change | Categorical | 4 | 51 | N/A | N/A | N/A | N/A | N/A | Low |
| app usage | Categorical | 2 | 0 | N/A | N/A | N/A | N/A | N/A | N/A |
| NRS itching at 3 months follow-up | Numeric | 11 | 109 | 2.42 | 2.7 | 1.0 | 0.0 | 10.0 | Low |
| DLQI score at 3 months follow-up | Numeric | 27 | 57 | 5.55 | 6.34 | 3.0 | 0.0 | 33.0 | Low |
| HADS anxiety score at 3 months follow-up | Numeric | 19 | 56 | 5.59 | 4.16 | 5.0 | 0.0 | 20.0 | Low |
| HADS depression score at 3 months follow-up | Numeric | 18 | 56 | 4.3 | 3.97 | 3.0 | 0.0 | 18.0 | Low |
| NRS pain at 3 months follow-up | Numeric | 11 | 109 | 2.15 | 2.58 | 1.0 | 0.0 | 10.0 | Low |

## Cross Validation Scores

| Fold | Cross Validation Score (AUC) |
| --- | --- |
| Fold 1 | 0.65668 |
| Fold 2 | 0.60599 |
| Fold 3 | 0.65323 |
| Fold 4 | 0.53704 |
| Fold 5 | 0.65046 |
