# Supplementary material for: Automated Machine Learning Analysis of Patients With Chronic Skin Disease Using a Medical Smartphone App: Retrospective Study
Source: J Med Internet Res. 2023 Nov 28;25:e50886. doi: 10.2196/50886 (PMC10716771; doi:10.2196/50886)
Supplement: Multimedia Appendix 4 [file jmir_v25i1e50886_app4.docx]

| **Itching development for 6 months** | **Pain development for 6 months** | **DLQI development for 6 months** | **App usage** | |
| --- | --- | --- | --- | --- |
|  |  |  | Regular Feature List | Reduced Feature List |
| Gender  Age  Body height  Body weight  BMI  nicotine  job type  Pre-existing conditions  leisure-time physical exercise (cut-off time of 2 hours per week)  Physical activity level at onset  NRS pain at onset  Onset disease activity  DLQI score at onset  HADS anxiety score at onset  HADS depression score at onset  DLQI categorical at onset  HADS anxiety categorical at onset  HADS depression categorial at onset  NRS pain at follow-up  DLQI score at follow-up  HADS anxiety score at follow-up  HADS depression score at follow-up  DLQI categorical at follow-up  HADS anxiety categorical at follow-up  HADS depression categorical at follow-up  pain development over 6 months  DLQI development over 6 months  HADS anxiety development over 6 months  HADS depression development over 6 months  had therapy change  app usage  app days in use  app average questions answered per day  Categorized app questions answered per day  total answered app questions  app average pain  app average Itching  app average DLQI total  app average compliance  app average tiredness/exhaustion  app average mood effected  app average activities effected by disease  app average morning stiffness  app average morning stiffness duration  average app sensitivity to touch or pressure (last 7d)  app average joint swelling (last 7d)  app average joint pain (last 7d)  app average DLQI total | Age  Body height  Body weight  BMI  nicotine  NRS itching at onset  Onset disease activity  DLQI score at onset  DLQI categorical at onset  NRS itching at follow-up  DLQI score at follow-up  HADS depression score at follow-up  DLQI categorical at follow-up  pain development over 6 months  had therapy change  app days in use  Categorized app questions answered per day  total answered app questions  app average compliance  app average morning stiffness duration  app average DLQI 6  app average DLQI 7  app average DLQI 7a  app average DLQI 8 | Age  Body height  Body weight  BMI  job type  NRS pain at onset  NRS itching at onset  Onset disease activity  HADS anxiety score at onset  HADS depression score at onset  NRS pain at follow-up  NRS itching at follow-up  HADS anxiety score at follow-up  HADS depression score at follow-up  pain development over 6 months  itching development over 6 months  DLQI development over 6 months  HADS depression development over 6 months  had therapy change  app days in use  Categorized app questions answered per day  app average mood effected  average app sensitivity to touch or pressure (last 7d) | Gender  Age  Body height  Body weight  BMI  nicotine  job type  Pre-existing conditions  leisure-time physical exercise (cut-off time of 2 hours per week)  Physical activity level at onset  NRS pain at onset  NRS itching at onset  Onset disease activity  DLQI score at onset  HADS anxiety score at onset  HADS depression score at onset  DLQI categorical at onset  HADS anxiety categorical at onset  HADS depression categorial at onset  NRS pain at 3 months follow-up  NRS itching at 3 months follow-up  DLQI score at 3 months follow-up  HADS anxiety score at 3 months follow-up  HADS depression score at 3 months follow-up  NRS pain at follow-up  NRS itching at follow-up  DLQI score at follow-up  HADS anxiety score at follow-up  HADS depression score at follow-up  DLQI categorical at follow-up  HADS anxiety categorical at follow-up  HADS depression categorical at follow-up  DLQI development over 6 months  HADS anxiety development over 6 months  HADS depression development over 6 months  pain development over 6 months  itching development over 6 months  app usage | Age  nicotine  comorbidities  onset disease activity  DLQI categorial at onset  DLQI categorical at follow-up  itching development over 6 months  HADS depression development over 6 months  app usage  NRS itching at 3 months follow-up  HADS anxiety score at 3 months follow-up |
