# Supplementary material for: Automated Machine Learning Analysis of Patients With Chronic Skin Disease Using a Medical Smartphone App: Retrospective Study
Source: J Med Internet Res. 2023 Nov 28;25:e50886. doi: 10.2196/50886 (PMC10716771; doi:10.2196/50886)
Supplement: Multimedia Appendix 6 [file jmir_v25i1e50886_app6.docx]

| **Target** | **Compared Models** | **Selected Model** | **LogLoss** | | | | **AUC** | | | **FVE Multinomial / Binomial*** | | |
| --- | --- | --- | --- | --- | --- | --- | --- | --- | --- | --- | --- | --- |
|  |  |  | **Validation** | **CV** | **Holdout** | | **Validation** | **CV** | **Holdout** | **Validation** | **CV** | **Holdout** |
| **Target 1: itching development for 6 months** | 78 | Light Gradient Boosted Trees Classifier (SoftMax Loss) (64 leaves) | 0.9302 | 1.1093 | | 0.9167 | 0.8096 | 0.7748 | 0.6374 | 0.2594 | 0.1642 | 0.0521 |
| **Target 2: pain development for 6 months** | 54 | Random Forest Classifier (Gini) | 1.1799 | 1.1561 | | 1.0976 | 0.7545 | 0.7684 | 0.7622 | 0.2047 | 0.2138 | 0.1780 |
| **Target 3: DLQI development for 6 months** | 27 | Random Forest Classifier (Gini) | 1.4065 | 1.3650 | | 1.4548 | 0.7072 | 0.7678 | 0.7260 | 0.0799 | 0.1557 | 0.1477 |
| **Target 4: app usage** | 216 | ENET Blender | 0.6509 | 0.6698 | | 0.6389 | 0.6567 | 0.6207 | 0.7232 | 0.0592 | 0.0305 | 0.0751 |
