# Supplementary material for: Automated Machine Learning Analysis of Patients With Chronic Skin Disease Using a Medical Smartphone App: Retrospective Study
Source: J Med Internet Res. 2023 Nov 28;25:e50886. doi: 10.2196/50886 (PMC10716771; doi:10.2196/50886)

Learning Curves (mean metric score / 95% Confidence Intervall)

Target 1: itching development for 6 months

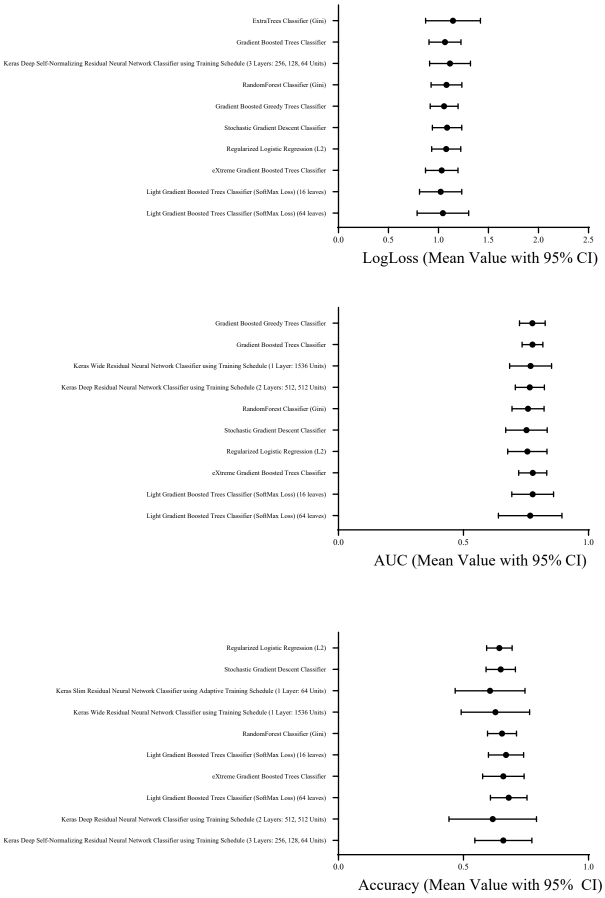

Target 3: DLQI development for 6 months

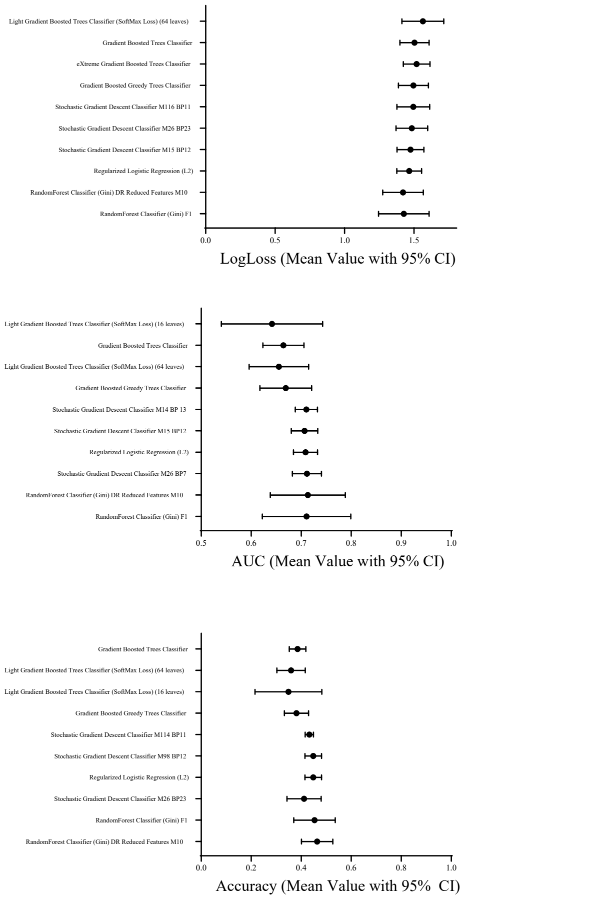

Target 2: pain development for 6 months

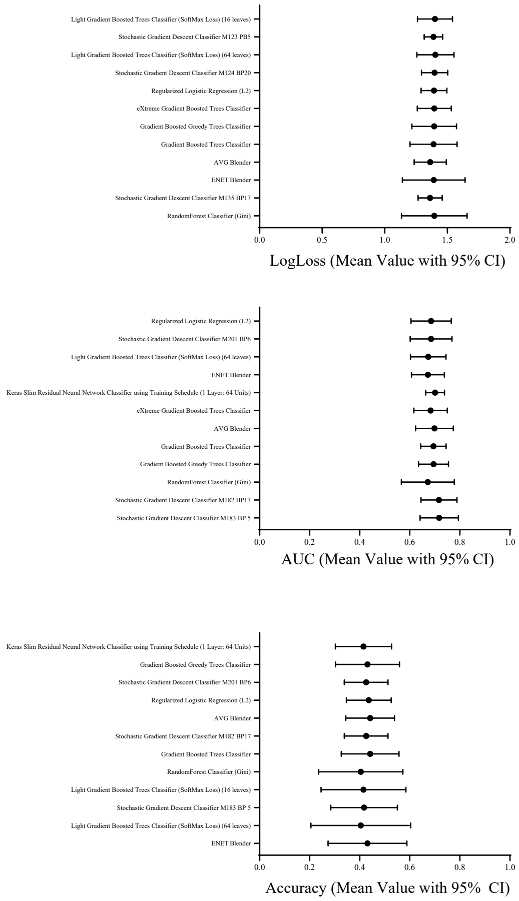

Target 4: app usage

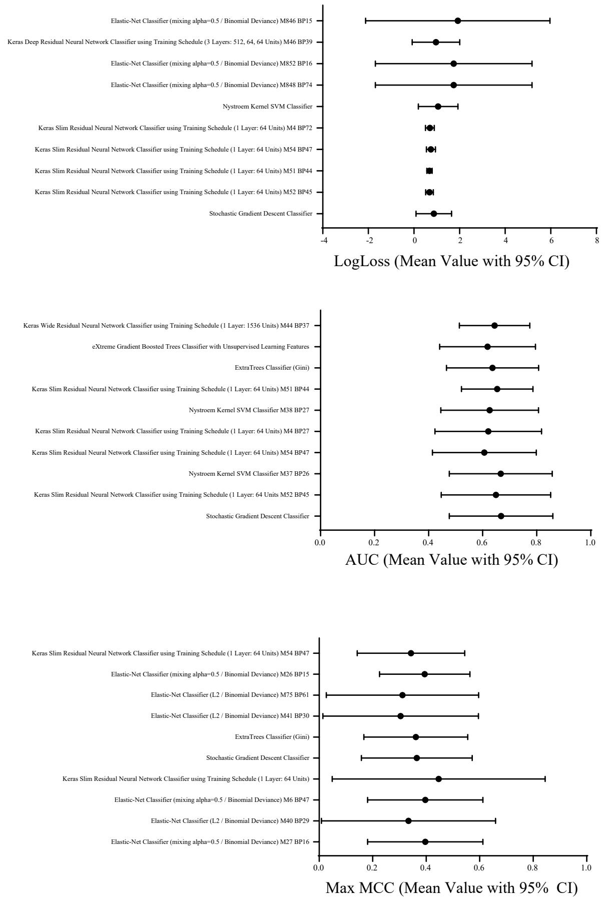

Supplement: Multimedia Appendix 7 [file jmir_v25i1e50886_app7.pdf]
